# Prevalence and Antimicrobial Resistance of Bacterial Uropathogens Isolated from Dogs and Cats

**DOI:** 10.3390/antibiotics11121730

**Published:** 2022-12-01

**Authors:** Sophie Aurich, Ellen Prenger-Berninghoff, Christa Ewers

**Affiliations:** Institute of Hygiene and Infectious Diseases of Animals, Faculty of Veterinary Medicine, Justus Liebig University Giessen, D-35392 Giessen, Germany

**Keywords:** urinary tract infection, prevalence, *E. coli*, enterococci, staphylococci, guidelines, MDR, nitrofurantoin (NIT)

## Abstract

Bacterial urinary tract infection (UTI) is a common diagnosis in companion animal practice and is one of the leading reasons for antimicrobial prescriptions. We analysed 1862 samples from the urinary tract of dogs and cats, submitted to a veterinary microbiological diagnostic laboratory in 2019 and 2020 in Germany. Susceptibility of 962 uropathogenic isolates to 15 antimicrobials, suggested as first- and second-line treatment options for UTI, was determined according to CLSI recommendations. Bacterial growth of uropathogens was detected in 43.9% of dog and in 38.5% of cat samples. *Escherichia (E.*) *coli* was the most frequently isolated pathogen (48.4%), followed by *Enterococcus* spp. (11.9%) and coagulase-positive staphylococci (CoPS; 11.5%). Females were more likely to exhibit a positive microbiological culture. Regarding first-line antibiotics, 93.4% of the most commonly isolated uropathogenic species were susceptible to the first-line antibiotics amoxicillin/clavulanic acid (AMC) and 87.6% to trimethoprim-sulfamethoxazole (SXT), while 76.1% showed decreased susceptibility to ampicillin (AMP). Multidrug resistance (MDR) was detected in 11.9% of *E. coli*, 50.4% of enterococci, and 42.7% of CoPS; 90.6% of these isolates were susceptible to nitrofurantoin (NIT). Our data indicate that empiric treatment of UTI with AMC or SXT could be recommended and is preferable to treatment with AMX. NIT should be considered for the treatment of MDR uropathogens.

## 1. Introduction

Bacterial urinary tract infection (UTI) is a commonly diagnosed disorder in companion animals. Approximately 14% of all dogs and 12–19% of all cats are suffering from this disease at least once in their lifetime [1,2,3]. UTI is among the main reasons for the prescription of antimicrobials, often without microbiological culture and antimicrobial susceptibility testing (AST) [4,5,6]. Moreover, in companion animals, the frequency of usage of highest priority critically important antibiotics (HPCIA), such as fluoroquinolones (FQs) and third-generation cephalosporins, is disturbingly high, particularly for the treatment of UTI [7,8,9,10]. In 2014, a survey of more than 3000 veterinary practitioners from 25 European countries revealed that up to 62% of the antibiotics prescribed for UTI in cats and 27% in dogs belonged to antimicrobial substances that have been classified as HPCIA by the World Health Organization (WHO) [11,12]. The WHO considers that the amount of antimicrobials used in the non-human domain has an impact on the occurrence of antimicrobial-resistant (AMR) bacteria in animals and by that on human exposure to these resistant bacteria [12]. In the last two decades, an increase of multidrug-resistant (MDR) bacteria implicated in UTI in companion animals has been reported [13]. This may not only lead to therapeutic failure but also to a public health concern in case of zoonotic pathogens or the transfer of AMR genes between animal and human pathogens [14,15]. The most common cause of UTI in dogs, cats, and humans is uropathogenic *Escherichia coli* (UPEC) [13,16,17]. In contrast to intestinal *Escherichia coli* strains, UPEC strains possess several virulence factors, which allow them to colonize the urinary tract and to enter the bladder epithelium [18]. Apart from UPEC, staphylococci, streptococci, enterococci, *Proteus* sp., *Klebsiella* sp., *Pseudomonas* sp., and *Enterobacter* sp. are commonly recognized as uropathogens [19].

A continuous surveillance on antimicrobial usage and antimicrobial resistance is necessary to identify trends and finally to update treatment guidelines. In addition, these data are necessary to frame risk management options on a wider level, such as restricted use, labeling, off-label use restriction or prohibition, and limited availability of antimicrobials by prescription only [12]. This is regarded as one of the most important tools to prevent an improper choice of antimicrobials and decrease the number of antimicrobial prescriptions [9,20].

The aim of our study was to provide phenotypic AMR data for bacterial pathogens involved in UTI in cats and dogs. Bacteria were cultivated from urine samples, differentiated to the species or genus level, and tested for their antimicrobial susceptibility. Here, we focused on antimicrobials that are advised for the treatment of UTI in cats and dogs, such as amoxicillin (AMX), amoxicillin/clavulanic acid (AMC), third-generation cephalosporins (3GC), FQs, nitrofurantoin (NIT), and trimethoprim-sulfamethoxazole (SXT) [21]. Furthermore, we aimed to identify possible risk factors for the presence of AMR bacteria in UTI, such as sex, age, previous antibiotic treatment, or comorbidities, based on metadata collected with a specifically designed questionnaire.

## 2. Results

### 2.1. Study Population

In a 12-months study period, 1862 samples from seven referral veterinary hospitals in Germany, including the Clinic for Small Animals of the Justus Liebig University (JLU) Giessen, and 52 primary care veterinary practices were submitted for routine microbiological diagnostics. Nearly two-thirds (62.7%) of the samples were provided by only four referral hospitals.

The majority of the samples (93.6%) consisted of urine (35.6% cystocentesis, 7.8% midstream voided samples, 6.1% catheter specimens, 50.5% unspecified sampling procedure) and, to a lesser extent (6.4%), of bladder swabs/bladder tissue, prostate swabs, Uricult tests, uroliths, and kidney swabs.

Overall, 1233 samples from 1013 dogs and 629 samples from 536 cats were submitted. Repeated samples were sent in 14.9% (dogs) and 12.9% (cats) of the cases.

A positive microbiological culture, which was defined as the isolation of at least one specific urinary bacterial pathogen as suggested by Barsanti et al. [19], or of growth of other bacteria in pure culture and/or in high numbers, was obtained in 43.9% of the samples from dogs. Of the 629 samples from cats, 38.5% revealed a positive microbiological culture. Positive cultures were 1.25 times more often obtained from specimens from dogs than from cats. Moreover, positive cultures were more likely observed from female than from male dog samples (*p* < 0.001). In samples from neutered cats (43.8%), a positive microbiological culture was detected more frequently (*p* < 0.001) than in the remaining samples from cats. In general, the mean age of animals with a positive microbiological culture (8.87 ± 4.69) was higher than the mean age of the total study population (8.02 ± 4.63) (Table 1).

**Figure 1:**
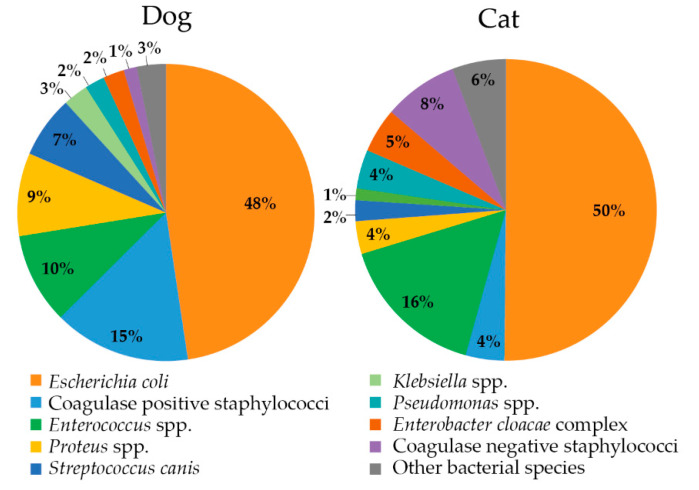
Prevalence of bacterial species recovered from the urinary tract of dogs (*n* = 697 isolates) and cats (*n* = 313 isolates). Other species: *Acinetobacter* spp., *Aerococcus viridans*, *Alcaligenes faecalis, Citrobacter freundii, Corynebacterium* spp., *Leclercia adecarboxylata*, *Morganella morganii*, *Pasteurella* spp., *Providencia stuartii, Pseudescherichia vulneris*, *Rahnella* spp., *Raoultella planticola*, *Serratia marcescens*, and *Stenotrophomonas* spp.

The most frequently isolated bacteria were *Escherichia* (*E.*) *coli* (dog: 47.6%/cat: 50.2%), *Enterococcus* species (spp.) (9.9%/16.0%), coagulase-positive staphylococci (CoPS) (14.9%/4.2%), *Proteus* (*P.*) spp. (9.0%/3.5%), *Streptococcus* (*Sc*.) *canis* (6.7%/2.2%), and coagulase-negative staphylococci (CoNS) (1.4%/8.0%) (Figure 1). The main identified enterococci species was *Enterococcus faecalis* (73.1%). Most of the CoPS (91.3%) were determined as *Staphylococcus* (*S*.) *pseudintermedius*. The genus *Proteus* was mainly composed of *P. mirabilis* (95.9%). Among the CoNS, 84.6% were assigned as *S. felis*, which was confined to samples from cats.

Among the positive microbiological samples, pure cultures containing a single bacterial species were isolated in 78.4% of the canine and 75.2% of feline the samples. In 16.6% of the canine and 19.4% of the feline samples, we determined two bacterial species. Three or more species were identified in 5.0% (range 1–5) and 5.4% (range 1–3) of the samples from dogs and cats, respectively. *E. coli* and CoPS were significantly associated with mono-infections (*p* < 0.001), whereas *Enterococcus* spp. were rather detected in mixed infections (*p* < 0.001), i.e., together with other specific uropathogens (Table 2).

Certain uropathogens were either associated with dog or cat samples. *Enterococcus* spp. (*p* = 0.0253), *Pasteurella* spp. (*p* = 0.0476), and CoNS (*p* < 0.001) were more likely detected in cats, while *Proteus* spp. (*p* = 0.0002), *Sc. canis* (*p* = 0.0007), and CoPS (*p* < 0.001) were more frequently obtained from dogs.

Furthermore, we could observe gender-related differences in the bacterial species distribution in both host species. CoPS (*p* = 0.0034) were more likely to be found in female dogs, and *Proteus* spp. appeared to be more often the cause of UTI in both female dogs (*p* = 0.0013) and cats (*p* = 0.0196).

### 2.2. Questionnaire

We requested all seven submitting referral veterinary hospitals and 52 primary care veterinary practices to contribute to this study by providing additional patient data. They were asked to complete a questionnaire regarding the animal’s signalment, the reason for its presentation to the veterinarian, clinical symptoms, comorbidities, type of specimen collection, and previous antimicrobial treatment (Supplementary Figure S1). Thereby, additional data were collected for 113 dogs (9.2% of the study population) and 48 cats (7.6%). Based on the replies to the questionnaires, the distribution of typical UTI symptoms was as follows: haematuria (43.0%), pollakiuria (26.1%), dysuria (21.1%), and stranguria (20.0%) [22]. The number of symptoms exceeds the number of animals, as some animals had multiple symptoms reported.

**Figure 2:**
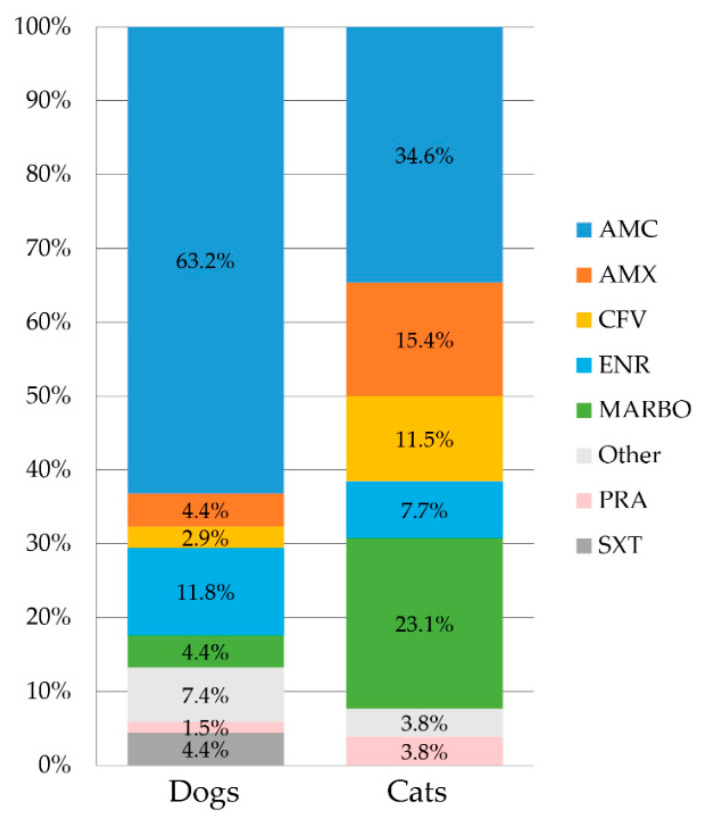
Percentages of antimicrobial substances used for the treatment of UTI in 43 dogs and 9 cats based on questionnaire data. Amoxicillin/clavulanic acid (AMC), amoxicillin (AMX), cefovecin (CFV), enrofloxacin (ENR), marbofloxacin (MAR), pradofloxacin (PRA), trimethoprim-sulfamethoxazole (SXT), and others (florfenicol, doxycycline, and unknown antibiotic agents).

Cats more likely suffered from dysuria than dogs (*p* = 0.0051). Antimicrobial treatment prior to microbiological culture was provided for 53.1% of the dogs and 45.8% of the cats. Up to three different antimicrobials per UTI treatment period were reported. In animals with available metadata, the most commonly used antibiotic was AMC (dogs 63.2%, cats 34.6%), and FQs were used in 34.6% of the cats and 17.6% of the dogs (Figure 2).

According to the completed questionnaires, 50.0% of the dogs and 66.0% of the cats presented with comorbidities in addition to clinical signs of UTI. In both animal species, the most commonly reported comorbidity was urolithiasis (dog 9.5%, cat 24.0%). Second most common comorbidity was renal dysfunction, including chronic kidney disease, acute kidney injury, glomerulopathy, and azotemia (dog 6.3%, cat 12.0%). In total, 7 out of 113 dogs (6.2%) presented with disorders of micturition such as incontinence and urine retention, and 5 out of 48 cats (10.4%) were diagnosed with diabetes mellitus. The spectrum of comorbidities in dogs was broader than in cats (*p* = 0.002). Detailed information about comorbidities is provided in Table S1.

### 2.3. Antimicrobial Susceptibility Data according to Broth Microdilution Testing

#### 2.3.1. Dogs

MIC data for 332 *E. coli* isolates from dogs are shown in Table 3. Regarding first-line antibiotics recommended for treatment of UTI, the susceptibility of *E. coli* isolates from dogs to AMC and SXT was high (94.9% and 88.8%), while it was lower (77.4%) to ampicillin (AMP), a surrogate for AMX [21]. Resistance to the second-line antibiotics cefovecin (CFV), enrofloxacin (ENR), and pradofloxacin (PRA) was less commonly detected (3.3–7.6%). According to the definition set by Magiorakos et al. (2012), forty-four (13.3%) of the isolates were considered as MDR, including two extensively drug-resistant (XDR) isolates (Table 4) [23].

Table S2 shows the MIC data distribution of 63 *Proteus* spp. from dogs. *Proteus mirabilis* is considered as intrinsically resistant to tetracycline and nitrofurantoin, whereas the two *P. vulgaris* isolates found in our study are additionally considered as intrinsically resistant to AMP and first-generation cephalosporins, according to CLSI Document M100 [24]. Both of them consequently exhibited an MIC of >8 mg/L (AMP) and >16 mg/L (CFX). In addition, both *P. vulgaris* isolates showed no resistance to any other tested antimicrobial. Most *Proteus* spp. from dogs were susceptible to β-lactam antibiotics, with the exception of AMP: 19 isolates exhibited an MIC of >16 mg/L and were classified as AMP-resistant (30.2%). For other common therapeutic agents such as FQs and SXT, resistance was found in 19.0% (ENR and PRA) and 22.2% (SXT) of the isolates.

Members of the *Enterobacter cloacae* complex (*EC*C) (Table S3) are considered as intrinsically resistant to AMP (MIC_50_ > 32 mg/L), AMC (MIC_50_ > 16 mg/L), and first-generation cephalosporins (MIC_50_ > 32 mg/L) [24]. Susceptibility to the remaining first- and second-line antibiotics ENR (81.3%), PRA (75.0%), SXT (87.5%), and CFV (62.5%) was decreased in the case of PRA and CFV.

Table S4 shows the MIC data distribution of 19 *Klebsiella* spp. isolates from dogs. Intrinsic resistance of *K. pneumoniae* (*n* = 14 isolates), *K. oxytoca* (*n* = 3), and *K. variicola* (*n* = 1) against AMP is clearly visible (MIC_50_ > 16 mg/L) [24]. *K. aerogenes* (*n* = 1) is also considered intrinsically resistant to AMC and to first-generation cephalosporins (both MIC_50_ > 16 mg/L) [24]. Susceptibility of *Klebsiella* spp. isolates was moderate to high for AMC and PRA (both 78.9%), ENR (84.2%), and CFV (89.5%). For SXT, all isolates were susceptible in vitro. Susceptibility to CFX appeared to be lower, with 36.8% of the isolates revealing a resistant phenotype. Almost one fifth (20.4%) of *Proteus* spp., *Klebsiella* spp., and *Enterobacter cloacae* complex isolates were classified as MDR, including three XDR isolates (*EC*C, *K. oxytoca*, *P. mirabilis*) and one pandrug-resistant (PDR) *EC*C isolate (Table 4).

Table S5 shows the MIC data distribution of 12 *P. aeruginosa* isolates. *P. aeruginosa* is considered as intrinsically resistant against AMP, AMX, AMC, CFX, CHL, CLI, ERY, SXT, TET, PEN, and OXA [24]. Accordingly, these antibiotics revealed high MIC values, except for SXT: only seven isolates had an MIC of ≥4 mg/L, while five isolates showed MICs of 1 to 2 mg/L. Because of the numerous intrinsic resistances, only 3GC, FQs, or aminoglycosides are deemed suitable for the treatment of UTI caused by this pathogen [25]. However, an animal-specific breakpoint is only given for GEN, and 83.3% of our isolates were identified as susceptible to GEN. For ENR, the cat-specific breakpoint for skin and soft tissue was used in accordance with CLSI document VET09 [26]. For PRA, in the absence of any animal or human breakpoint, the breakpoint set by the manufacturer was applied (S ≤ 0.25, I = 0.5–1, R ≥ 2). Based on that, 66.7% of the *P. aeruginosa* isolates were interpreted as susceptible to ENR and 16.6% as susceptible to PRA. Notably, the MICs of CFV (≥4 mg/L) indicated non-susceptibility for all 12 *P. aeruginosa* isolates. Four of the isolates showed an XDR pattern, and one isolate was resistant to all tested antimicrobials and therefore categorized as PDR (Table 4).

In Table 5, MIC data of the second most isolated species in dogs, namely CoPS, are shown. More than 90% of the 104 isolates were susceptible to first- and second-line antibiotics for treatment of UTI with MIC_90_´s of 0.125 to 0.5 mg/L. Only for AMP, susceptibility was at 48.1%, when in absence of dog-specific breakpoints, cat-specific breakpoints were applied in accordance with CLSI document VET09 [26]. The distribution of AMP MICs was significantly different from the other antibiotics: 50% of the values were at 0.5 mg/L, and the MIC_90_ was substantially elevated at 8 mg/L. Phenotypic resistance to oxacillin (OXA) was observed in six canine isolates (four *S. pseudintermedius*, one *S. aureus*, and one *S. schleiferi* ssp. *coagulans*). These isolates were considered resistant to all other β-lactam agents. An MDR phenotype was observed in 41 (39.4%) isolates, including two XDR *S. pseudintermedius* isolates (Table 4).

The MIC data distribution of 66 enterococci is shown in Table S6. According to CLSI document M100, *E. faecalis* and *E. faecium* are intrinsically resistant to cephalosporins, aminoglycosides (low-level resistance), CLI, and SXT, which is consistent with the obtained MIC data [24]. Therefore, only the results for AMC, AMP, ENR, and PRA are further described, as they are relevant for the treatment of UTI. More than 90% of the enterococci isolates were susceptible to AMC and AMP. Susceptibility to ENR (81.8%) and PRA (80.3%) was even lower. Thirty isolates (44.8%) revealed an MDR phenotype, and four of five *E. faecium* isolates were even categorized XDR (Table 4).

*Streptococcus canis* isolates showed high susceptibility (>90%) to all antimicrobial substances relevant for the treatment of UTI (Table S7).

#### 2.3.2. Cats

The MIC distribution of 157 *Escherichia coli* isolates from cats is shown in Table 6. The majority of *E. coli* isolates (>90%) were susceptible to most antimicrobials tested, when applying dog-specific breakpoints for feline *E. coli* to AMP, CFX, PRA, and ENR (taking into account that neither cat- nor human-specific breakpoints are available). This is in concordance with the recommendations in CLSI document VET09 [26]. Only in case of AMP, the percentage of susceptible isolates was lower (76.4%), corroborating with the elevated MIC data (MIC_90_ ≥ 16 mg/L). Fourteen isolates (8.9%) were classified as MDR (Table 4).

Other *Enterobacterales* than *E. coli* were not as common as in dogs. All eleven *Proteus* spp. isolates were AMC-susceptible. In total, 81.8% of *Proteus* remained susceptible to AMP and SXT. Moreover, susceptibility to second-line antibiotics, including ENR, CFX, and CFV, was high (90.9%) (Table S8). As members of the *Enterobacter cloacae* complex are intrinsically resistant against most β-lactam antibiotics, the only effective first-line antibiotic is SXT. Our isolates showed moderate susceptibility (80.0%) to SXT. Lower susceptibility was found for ENR and CFV (both 73.3%), while susceptibility to PRA remained high (86.7%) (Table S9). Phenotypic resistance of *Proteus* spp., *Klebsiella* spp., and *EC*C to 3GC was observed in three feline isolates (10%). Almost one-fourth (23.3%) of the isolates from cats showed an MDR pattern. Two *EC*C isolates were XDR, with only CHL and TET (both MIC of 8 mg/L) remaining as possible treatment options (Table 4).

In cats, only 50.0% of the 12 *P. aeruginosa* isolates were susceptible to FQs (Table S10). Like for dogs, there is no animal-specific nor human-derived breakpoint for PRA for UTI, so the manufacturer-derived breakpoint was applied. In the case of ENR, the manufacturer-derived breakpoint and cat-specific breakpoint for skin and soft tissue (SST) were identical and were applied. As for gentamicin, dog-specific breakpoints were applied in accordance with VET09 [26]; 83.3% of the isolates were susceptible. An XDR or PDR phenotype was observed in four and two isolates, respectively.

The MIC data distribution of 13 CoPS is shown in Table 7. In contrast to dogs, susceptibility of CoPS from cats appeared to be poor. It ranged from 15.4% (AMP) to 75% (CFV). MIC data were elevated accordingly: MIC_90_ for SXT and ENR was ≥4 mg/L, for CFV 8 mg/L, for AMP ≥ 16 mg/L, and for CFX ≥ 32 mg/L. Nine isolates (69.2%) were MDR, with three *S. pseudintermedius* showing an XDR pattern. Phenotypic resistance to OXA was observed in three feline *S. pseudintermedius* isolates.

MIC data of *Enterococcus* spp., the second most detected bacteria in cats (*n* = 50 isolates), are shown in Table S11. As there are only human-derived breakpoints for AMP, AMC, and FQs, manufacturer-derived breakpoints were applied. Like in dogs, susceptibility to β-lactam antibiotics was higher (91.8%) than to FQs (84% ENR, 76% PRA). Twenty-nine isolates (58%) were MDR, including all three *E. faecium* isolates. Two of these isolates were also considered XDR and were only susceptible to PEN and CHL (Table 4).

All but one of twenty-three CoNS from cats were identified as *S. felis*. Except for AMP (82.6%), all other antimicrobials important for the treatment of UTI were highly active against this pathogen (95.7–100%) (Table S12). None of the *S. felis* isolates presented an MDR pattern nor showed resistance to OXA.

### 2.4. Antimicrobial Susceptibility Data according to Standard Disk Diffusion Test

To determine bacterial resistance to NIT, inhibition zone diameters of 325 *E. coli* isolates, 83 CoPS, 92 enterococci, 32 streptococci, 19 *EC*C, and 14 *Klebsiella* spp. were determined by agar disk diffusion test according to CLSI document VET01 (Table 8) [28]. CoPS and *Sc. canis* were 100% susceptible in both animal species. High susceptibility was also determined for enterococci from dogs (90.9%) and cats (83.8%). Among *Enterococcus* spp., all *Enterococcus faecium* isolates showed resistance to NIT.

Of the Gram-negative bacteria, *E. coli* showed high rates of susceptibility, accounting for 92.5% in dogs and 91.9% in cats, while *Klebsiella* spp. and *EC*C had much lower susceptibility rates.

Of all MDR isolates tested (*n* = 91), 94.5% were susceptible to NIT.

### 2.5. Phenotypic Screening for ESBL Producing Isolates

Phenotypic resistance of *Enterobacterales* to 3GC was observed in 17 (3.9%) canine and 8 (4.2%) feline isolates. VITEK^®^2 testing confirmed an ESBL phenotype for 71.4% (dog) and 66.7% (cat) of the isolates tested. Of these, 68% were obtained from referral hospitals. Three of the *E. coli* isolates originated from one dog that was sampled at different times; each time, a mono-infection with an ESBL-producing *E. coli* was detected (Table 9).

## 3. Discussion

The aim of our study was to determine phenotypic AMR data for bacterial pathogens of dogs and cats suffering from UTI and to collect metadata to identify possible risk factors for UTI in these animals.

Consistent with the results from previous studies, significantly more samples from female than from male dogs yielded bacterial growth (*p* < 0.001), although the overall number of samples submitted from males was higher [30,31,32,33]. Likewise, in cats, the number of samples sent and thus the number of animals presenting with symptoms of lower urinary tract infection (LUTD) was much higher in males than in females, although samples from females more often yielded bacterial growth. This impression also emerged in other European studies: while male cats were more likely to have idiopathic LUTD, uroliths, or urethral plugs, evidence for an etiological role of bacteria as the cause of these symptoms was more often provided in female cats [2,3,34,35]. Additionally, in our study, a positive microbiological culture was significantly more often found among samples from neutered dogs (*p* = 0.023) and cats (*p* < 0.0001) compared with non-neutered animals.

UTI is typically caused by bacteria invading the urinary tract from the colon or skin [22,36]. It is commonly suggested that an increased detection of bacteria in female urethral samples is mainly due to anatomical reasons, i.e., the length of the urethra and the short distance between the urethral meatus and anus [22,37]. Moreover, the surgical removal of the uterus and ovaries (ovariohysterectomy) might impair the self-defence mechanisms of the bladder [38,39]. With the cessation of estrogen release, the production of mucopolysaccharides, which prevent bacterial pathogens from adhering to the bladder epithelium, is no longer under control. For males, an antimicrobial effect of prostatic secretions has been noted [40].

Feline lower urinary tract disease (FLUTD) is mostly due to feline idiopathic cystitis (FIC) or urolithiasis and less frequently caused by bacterial infections [34]. Bacterial UTI has been described as the cause of FLUTD in only 7.8–18.9% of cases studied in Europe [2,3,34,35,41]. In our study, 38.5% of the feline samples yielded bacterial growth. This could be explained with the higher percentage of female cats in our study (40%) compared to the above-mentioned studies (13–26%).

Dorsch et al. (2014) reported that a majority of cats suffering from FLUTD had relevant predisposing comorbidities [2]. This was in line with our observations: 72.9% of the attending veterinarians reported a comorbidity in the questionnaire. Almost 23% of the animals suffered from urolithiasis, a higher percentage than previously detected [2,3,35,41]. Only Gerber et al. (2005) reported a similar percentage (22%) of cats with uroliths, although it must be considered that nearly 90% of their study population consisted of males [34].

A limitation of our study was the inability to distinguish between asymptomatic bacteriuria (ASB) and urinary tract infection. According to current guidelines, ASB is defined as “the presence of bacteria in urine as determined by positive bacterial culture from a properly collected urine specimen, in the absence of clinical evidence of infectious urinary tract disease” [21] (p. 17). Since most veterinarians did not report back whether the animal had clinical signs, a bias in our results due to cases of ASB cannot be ruled out.

The incidence of polybacterial (22.6%) versus monobacterial (77.4%) infections in dogs and cats was consistent with other studies [32,42,43]. Interestingly, mixed infection with more than three pathogens at the same time only occurred in dogs. Also in accordance with other studies worldwide, the most prevalent bacterium isolated from UTI in cats and dogs was *E. coli*, followed by *Enterococcus* spp., CoPS, and *Proteus* spp. [13,17,30,31,32,44,45,46,47,48,49]. CoPS, *Proteus* spp., and *Sc. canis* were preferentially found in dogs, whereas *Enterococcus* spp. and *S. felis* were more frequently found in cats (all with statistical support). Except for *E. coli*, a predilection of a bacterial species for either dogs or cats has also been shown in other studies that compared feline and canine uropathogens [17,48,49,50]. However, a separation between CoPS and CoNS was rarely performed. If so, *S. felis* was exclusively found in cats, and CoPS, especially *S. pseudintermedius*, were significantly more often detected in dogs [43]. Recent studies showed that the CoPS species *S. pseudintermedius* is not part of the physiological skin microbiota of cats [51,52,53]. Given the fact that one of the most common causes of UTI is ascending infection from skin, this may explain the much lower prevalence of *S. pseudintermedius* as a uropathogen in cats compared with dogs. The majority of isolated enterococci species consisted of *E. faecalis* (71.8%), followed by *E. faecium* (7.6%) and seven other enterococci species (20.5%). The fact that *E. faecalis* is more abundant can be explained by its increased virulence: it has, for example, a more pronounced ability to form biofilms, so that it adheres more strongly to biotic surfaces and is thus protected from urine flushing and local endogenous defences and antimicrobial substances [54,55,56]. *E. faecium*, meanwhile, is much more resistant: all *E. faecium* isolates presented an MDR pattern.

For the treatment of uropathogenic *E. coli*, our results showed a reduced susceptibility to AMP in both animal species, which agrees with several other studies [30,31,44,57,58]. Likewise, most of our and previously published strains were found to be AMC-susceptible according to CLSI document VET01S. For the other antimicrobials commonly used to treat UTI caused by UPEC, resistance of *E. coli* was low in both animal species.

The second most isolated Gram-negative pathogen was *Proteus* spp. In contrast to cats, where susceptibility to AMP, SXT, ENR, and PRA was moderate to high (81.8–90.9%), dog isolates showed reduced susceptibility to AMP (69.8%), SXT (77.8%), ENR (77.8%), and PRA (73.0%). AMC continued to prove a good option for treatment of UTI with 96.8% of isolates being AMC susceptible.

Except for AMP (48.1%, cat-specific breakpoints applied in accordance with CLSI document VET09), susceptibility of canine CoPS to antibiotics recommended for the treatment of UTI was > 90%. In cats, however, the results are more worrying: even though only 4.2% CoPS were identified among all feline UTI cases, these isolates showed more or less reduced susceptibility to almost all antibiotics, including AMC (38.5% susceptible isolates), AMP (15.4%), SXT (53.8%), and the FQs (46.2%). Only for CFV, two-thirds of the isolates were still susceptible (75.0%). Almost 70% of feline CoPS exhibited an MDR phenotype. By comparing MIC_50_ and MIC_90_, feline CoPS proved to be significantly more resistant to all tested antibiotics (excluding SXT: *p* = 0.0504) than canine CoPS (AMP *p* = 0.0026, AMC *p* < 0.001, CFX *p* = 0.0023, CFV *p* < 0.001, ENR *p* = 0.0007, GEN *p* < 0.001, and PRA *p* = 0.0002). The lower susceptibility of feline staphylococci compared with canine isolates is consistent with recent studies. In a study from Spain, almost 46% of CoPS isolates recovered from cats with signs of UTI were categorized as MDR, and 23% were XDR [49]. Marques et al. (2018) reported 12 times less CoPS in cats than in dogs. However, nearly all (91%) methicillin-resistant *S. aureus* (MRSA) isolates detected in their study originated from cats [13]. A study from Italy revealed resistance of three *S. pseudintermedius* isolates from cats to all UTI-relevant antimicrobials and resistance of two *S. aureus* isolates, also from cats, to all β-lactam antimicrobials, as well as reduced susceptibility to FQs [20].

**Figure 3:**
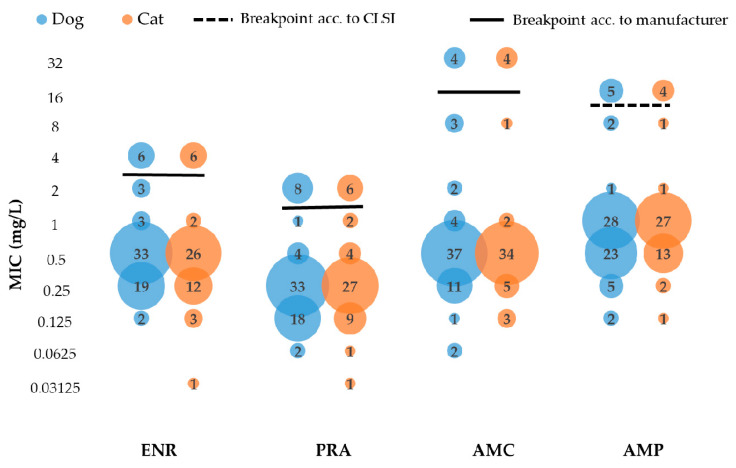
Distribution of MIC data of 66 canine and 50 feline enterococci for the antimicrobials ENR, PRA, AMC, and AMP. acc. = according.

In *Enterococcus* spp. isolates from our study, antimicrobial susceptibility differed only slightly between dog and cat isolates. As more than 90% of the isolates were susceptible to AMC and AMX, these drugs should be considered as agents of choice. Although in 87.5% of the isolates MIC values for ENR and PRA ranged between 0.03125 and 1 mg/L, FQs cannot be recommend for the treatment of enterococcal UTI because of the reduced efficacy of FQs against enterococci in vivo [29]. The validity of these results is limited by the lack of animal species- or even human-derived breakpoints for enterococci, as only for AMP is a human-derived CLSI breakpoint given. As shown in Figure 3, the manufacturer’s breakpoint interpretation of susceptible, intermediate, and resistant (S/I/R) appears reasonable. The MIC data distribution is bimodal, suggesting there might be a resistant subgroup of isolates [59]. Naturally, enterococci are not the only bacteria with a bimodal distribution. For example, the distribution of ENR- and PRA-MIC data of *E. coli* also shows this distribution (Table 6).

The percentage of *P. aeruginosa* was significantly higher in samples from animals with repeated samples (four or more) during the study period (10.5%) compared to the entire sample pool (2.8%). Repeated submission of samples could suggest recurrent UTI in these animals. Indeed, increased isolation rates of *P. aeruginosa* in samples from recurrent UTIs are consistent with previous studies, as they have many intrinsic resistances and are often MDR (41.7% in our study) [32,60]. In case of *P. aeruginosa* infection, FQs are considered the drugs of choice. Notably, our data showed reduced susceptibility for a considerable part of these isolates (dog, ENR 66.7%, PRA 16.6%; cat, ENR and PRA 50%). The isolates showed a much higher susceptibility to gentamicin; however, the use of aminoglycosides in UTI is limited: they are potentially nephrotoxic, must be administered parenterally on a daily schedule, have significantly decreased activity in acidic urine, and are potentially inactivated by purulent debris [61].

Overall, the number of MDR isolates was significantly higher in Gram-positive cocci (*Enterococcus* spp., 50.4%; CoPS, 42.7%; *Sc. canis*, 11.3%; *S. felis*, 0%; total, 37.2%) than in Gram-negative species (*E. coli*, 11.9%; *Proteus* spp., 18.9%; *EC*C, 25.8%; *Klebsiella* spp., 21.7%; *P. aeruginosa*, 41.7%; total, 14.8%) (*p* < 0.001). Although *E. coli* was the most frequently detected uropathogen, the number of MDR isolates was low compared to other *Enterobacterales*, which is consistent with the results from other studies [13,49]. Among Gram-positive cocci, particularly *Enterococcus* spp. isolates showed a high number of MDR bacteria. The frequency of MDR enterococci from the urine of dogs and cats varied largely among different studies (20–100%) but is partially comparable to our findings [31,43,46,54,62,63]. It should be noted that most veterinarians would rather submit urine specimens in case antimicrobial treatment without prior urine culture and antimicrobial susceptibility data (empiric therapy) failed or if the patient suffers from recurrent infection (either relapsing infection or reinfection) [64]. In other words, the corresponding isolates have most likely been previously exposed to antimicrobials and might have developed resistance. This is supported by our data as according to the replies to the questionnaire: 47.7% of the animals have undergone antimicrobial treatment prior to sampling and culturing of bacteria [65,66]. Nonetheless, unlike with livestock, MDR infections are more common in companion animals, and the treatment of such infections is challenging. In particular serious cases, it may even create the need for off-label use of antimicrobials reserved for humans [67].

International guidelines for the management of UTI have been available from the International Society for Companion Animal Infectious Diseases since 2011 and in a current iteration (2019). Recommendations for the treatment of UTI are also available in several national guidelines online or in textbook form [21,61,68,69,70,71,72,73]. In the vast majority of these documents, a classification is made between sporadic bacterial cystitis (formerly termed as uncomplicated cystitis) and recurrent cystitis (complicated cystitis). For the treatment of sporadic cystitis, first-line antibiotics such as AMX or SXT are recommended. The use of a β-lactamase inhibitor such as clavulanic acid should only be used for empirical therapy when regional susceptibility data demonstrate a high prevalence for AMX resistance but susceptibility for AMC [21]. If first-line antibiotics are not appropriate according to AST results, the second-line antibiotics NIT, FQs, or 3GC can be administered. However, these antimicrobial agents should be used with care regarding their importance in human health. In case of recurrent bacterial cystitis, AST should always be performed; initial antimicrobial therapy with first-line antibiotics or administration of NSAIDs is indicated while awaiting AST results. Antimicrobial treatment should be adjusted depending on AST results [22].

According to the questionnaire, most animals in our study received prior treatment with AMC. In the year 2019, Weese et al. stated: “If the expected incidence of treatment failure to a given antimicrobial increases, an alternate antimicrobial should be considered” [21] (p. 10). The Infectious Diseases Society of America and European Society for Microbiology and Infectious Diseases guidelines for humans for acute uncomplicated urinary tract infections do not recommend the use of AMP or AMX, given the high resistance (approx. 30%) determined in human isolates. Other antibiotics, such as SXT, should be withdrawn from use when resistance prevalence has increased above a threshold of 20%, i.e., in this case, the agent is no longer recommended for treatment [74]. Considering that many *Enterobacterales* are intrinsically resistant to AMX and that, except for enterococci and *Sc. canis*, susceptibility was considerably lower to AMP than to AMC (76.1% vs. 93.4%, Table S13), the use of AMX as a first-line antibiotic for UTI should be reconsidered. Instead, direct use of AMC should be taken in consideration.

SXT was used to treat UTI in only 4.4% of the study cases and only in dogs. While the use of SXT in veterinary medicine was widely described in the 1970s, 1980s, and 1990s, the use of this antimicrobial agent declined in the 2000s due to the increasingly observed side effects [68,75,76]. Current guidelines recommend the use of SXT but also point out a shorter usage of antimicrobials in the treatment of UTI [21]. Since most side effects occur only after a longer treatment period (average of 12 days), SXT is a good alternative to AMX as a first-line antibiotic for sporadic bacterial cystitis when administered for a short period of time [76]. This is shown in the study by Clare et al. (2014) and is consistent with our findings regarding high susceptibility of *Enterobacterales*, despite *Proteus* spp., to this drug [77]. However, if the animal suffers from recurrent bacterial cystitis, short-term sulphonamide treatment may not be sufficient, as high clinical remission rates were previously reported for such cases [77].

In most of the current guidelines, first-generation cephalosporins are not recommended for first- or second-line treatment, but in Germany, CFX is approved for the treatment of cystitis for susceptible *Enterobacteriaceae*, streptococci, and staphylococci for dogs and cats. According to AST results, most *Enterobacterales* showed susceptibility of more than 90% to this drug. The susceptibility results for streptococci and staphylococci in dogs support the choice of CFX for canine bacterial UTI. However, it must be taken into consideration that in cats, enterococci were the most commonly detected Gram-positive bacteria and that these are intrinsically resistant to CFX. Therefore, when Gram-positive cocci are detected in the cat, the use of CFX should be discouraged.

Nitrofurantoin is recommend by current guidelines as a second-line antimicrobial in sporadic bacterial cystitis [21]. However, due to its toxicity and poor pharmacokinetic properties, it should only be considered in cases of UTI caused by MDR bacteria [78,79,80,81]. The excellent efficacy of NIT is also supported by our data, as 94.5% of the MDR isolates identified in this study were susceptible to this drug. Only for *Klebsiell*a spp., *EC*C, and *Enterococcus faecium*, treatment with NIT cannot be recommended due to high resistance rates in our study. It should also not be applied in infections caused by *Proteus* spp. and *Pseudomonas* spp., as these bacteria are intrinsically resistant to NIT [82]. In contrast to earlier reports, the macrocrystalline formulation of NIT prescribed today rarely causes gastrointestinal side effects [80,83]. Clinical treatment with NIT also requires excellent compliance from the owner, as the drug must be administered every 8 h due to its low plasma concentration [21,78,83]. It has to be used as an off-label drug under the Cascade principle, as there is no equivalent product licensed for veterinary usage in Germany.

Prudent use of antibiotics, particularly of drugs that are critically important for humans, such as FQs and 3GC, is one essential element in the global fight against AMR. Our data, although limited to a small study population, suggest that empiric UTI therapy was often performed with ENR, MAR, or CFV. This was particularly the case in cats, which was previously also noted by Weese et al. (2021) [84,85]. FQs and 3GC are classified by the European Medicines Agency (EMA) for “restrict use” in animals to mitigate public health risk, and the WHO classifies them as HPCIA [12,86]. They are also first-line agents for treatment of pyelonephritis and other infections with MDR bacteria in veterinary medicine [21]. Prescription of these antimicrobials is very common in cats [7,9,84,87,88,89]. This could likely be explained with convenient application. Whereas AMX, AMC, or SXT must be given orally every 12 h, FQs require this only every 24 h, and a single injection with CFV is sufficient for up to 14 days [21]. According to a previous study evaluating electronic medical records, the most common reason for prescribing CFV was the inability to treat cats orally [10].

The problem of overprescribing is not limited to cats. Several studies observed that antimicrobial therapy is initiated far too often in animals that are presenting urinary symptoms [5,9,90]. According to a Danish study, verification of the suspected UTI diagnosis was achieved in 80.1% of the cases by microscopy and in only 56% by microbiological culture [91]. This is in agreement with the results of a Swiss study, which reported that in only 40% of all FLUDT cases was a diagnostic work up with microbiological culture initiated [90]. This kind of diagnostic approach favours the selection of MDR bacteria and increases the risk of transmission of MDR bacteria between companion animals and humans [14,92,93].

## 4. Materials and Methods

### 4.1. Bacterial Isolates

Samples collected for routine microbiological diagnostic from dogs and cats treated in referral veterinary hospitals and primary care practices were investigated for bacterial growth during a twelve-month study period (November 2019 to November 2020). Only samples originating from the urinary tract, such as urine (either midstream voided or obtained by catheterization or by cystocentesis), bladder epithelium, bladder swabs, prostate swabs, and uroliths were included in this study. Species, age, and sex were determined from the submission form that was sent by all participating clinics and primary care practices along with the samples, either electronically by a practice management software or as printed document.

### 4.2. Questionnaire

By means of a questionnaire (Figure S1), the participating veterinarians were asked to provide detailed information about the sample origin. This questionnaire enquired about animal features, such as age and sex of the animal and why it was presented to the veterinarian. Common clinical signs associated with UTI, such as stranguria, pollakiuria, or haematuria, were evaluated [19,22,94]. Comorbidities, the method of urine collection, and previous antimicrobial treatment were also recorded. The questionnaire, together with a privacy statement, was provided to each practice by E-mail. The study participants were asked to return the completed questionnaire together with the corresponding sample.

### 4.3. Isolation and Identification of Bacterial Isolates

For microbial investigation, the samples were streaked out on standard nutrient agar (Oxoid, Wesel, Germany) containing 5% defibrinated sheep blood and on water-blue metachrome-yellow lactose agar according to Gassner (sifin diagnostics gmbh, Berlin, Germany). Plates were incubated under aerobic conditions at 37 °C and checked for growth after 24 and 48 h. For bacterial enrichment, the samples were additionally cultivated in standard I nutrient broth (E. Merck, Darmstadt, Germany) for 24 h at 37 °C and streaked on 5% sheep blood agar and Gassner agar. Species identification was performed with matrix-assisted laser desorption time-of-flight mass spectrometry (MALDI-TOF-MS, Microflex, LT, V3.3.1.0, Bruker Daltonics, Bremen, Germany) according to the manufacturer’s instructions. All isolates were stored in 30% glycerol in Brain Heart Infusion Broth (Oxoid, Wesel, Germany) at −70 °C.

### 4.4. Inclusion Criteria for Presumptive Uropathogens

A bacterium was considered uropathogenic if (a) it was detected in pure culture or (b) in high numbers and (c) if the animal showed clinical symptoms of UTI or (d) if the animal was previously treated with antibiotics due to UTI [19,26,95]. Growth was estimated semi-quantitatively: isolated bacterial growth, 1–5 colonies, (+); bacterial growth, 6–50 colonies, +; moderate bacterial growth, 51–200 colonies, ++; strong bacterial growth, >200 colonies, +++. In case mixed cultures or low bacterial counts were determined from urine that was not obtained by cystocentesis, only bacteria listed in Table 10 were considered as uropathogens.

### 4.5. Antimicrobial Susceptibility Testing and Definition of Multidrug Resistance (MDR)

Presumptive uropathogenic isolates were tested for their antimicrobial susceptibility by broth microdilution as recommended by CLSI document VET01, using the MultiscanTM FC Microplate Photometer (Thermo Fisher Scientific, Dreieich, Germany) [28].The evaluation was performed with the software Merlin Micronaut-S, small animal layout 3 (Merlin Diagnostika GmbH, Bornheim-Hersel, Germany). According to a test design of a national working group on veterinary medical infectious diagnostic (AVID, Arbeitskreis Veterinärmedizinische Infektionsdiagnostik of the German Veterinary Society), the following 14 antimicrobial substances were included for MIC determination: ampicillin (AMP), amoxicillin/clavulanic acid (AMC), cephalexin (CFX), cefovecin (CFV), clindamycin (CLI), chloramphenicol (CHL), enrofloxacin (ENR), erythromycin (ERY), gentamicin (GEN), oxacillin (OXA), penicillin (PEN), pradofloxacin (PRA), trimethoprim-sulfamethoxazole (SXT), and tetracycline (TET) [27]. The results were interpreted according to CLSI document VET01S [28,29]. In accordance with CLSI document VET09, in the absence of species-specific breakpoints, canine breakpoints were applied to cats and conversely; in the absence of breakpoints for *Enterobacterales*, the species-specific breakpoints for *E. coli* were applied; in the absence of breakpoints for UTI, species-specific breakpoints derived from skin and soft tissue were used [26]. If animal breakpoints were not available, human-derived breakpoints from CLSI document M100 were applied [24]. In cases of neither animal- nor human-derived breakpoints, the breakpoints set by the manufacturer were applied, as these are also applicable in routine diagnostics. Furthermore, the concentration of an antimicrobial that is required to inhibit bacterial population growth by 50% or 90% (MIC_50_ and MIC_90_) was calculated based on the measured MIC values. *Escherichia coli* ATCC 25922, *Staphylococcus aureus* ATCC 29213, *Enterococcus faecalis* ATCC 29212, and *Pseudomonas aeruginosa* ATCC 27853 served for quality control [29].

In case resistance to the 3GC CFV was detected in members of *Enterobacterales*, a verification for ESBL-producing isolates was performed: the isolates were tested using the VITEK^®^2 compact 15 with the VITEK^®^2 system (V9.03.2, bioMérieux, Nürtingen, Germany) using the ESBL test panel included in the AST card GN97 (bioMérieux, Nürtingen, Germany). In detail, broth microdilution was performed with Mueller Hinton broth (Oxoid, Wesel, Germany) containing serial twofold dilutions of ceftazidime and cefotaxime (both 0.5 mg/L) with and without clavulanic acid at a fixed concentration of 4 mg/L for Group I species (without inducible chromosomal AmpC β-lactamases, *E. coli*, *Klebsiella* spp., *Proteus mirabilis*). For Group II species (*Enterobacteriaceae* with inducible chromosomal AmpC β-lactamases, *Enterobacter* spp.), cefepime (1 mg/L) with and without clavulanic acid (10 mg/L) was used. The test was assessed as positive if the MIC of any of the cephalosporins in combination with clavulanic acid was reduced ≥ 8-fold compared with the MIC of that cephalosporin alone according to EUCAST [96].

The susceptibility for NIT was tested by standard disk diffusion according to CLSI criteria [97], using Mueller Hinton Agar (Oxoid, Wesel, Germany) and NIT 300 µg discs (Mast Group Ltd., Bootle, UK), and was interpreted according to CLSI criteria [28]. As interpretive criteria were only available for *Enterobacteriaceae,* staphylococci, enterococci, and streptococci, only members of this group were tested. *Escherichia coli* ATCC 25922 served for quality control purposes.

An isolate was defined as multidrug-resistant (MDR) if it showed non-susceptibility (defined as resistant, intermediate, or non-susceptible) to at least one agent out of three or more antimicrobial classes according to Magiorakos et al. (2012). An extensively drug-resistant (XDR) isolate was defined as non-susceptible to at least one agent in all but two or fewer antimicrobial categories. Pandrug-resistance (PDR) was defined as non-suceptible to every tested antimicrobial [23]. Intrinsic resistances were excluded from this definition. *E. coli* was categorized as MDR according to its non-susceptibility to GEN, CFX, CFV, AMC, CHL, FQs (ENR and PRA), SXT, TET, and AMP, for *Proteus* spp., the substances GEN, CFX, CFV, AMC, CHL, FQs (ENR and PRA), SXT, and AMP, for *EC*C and *K. aerogenes*, GEN, CFV, CHL, FQs (ENR and PRA), SXT, and TET were used, and for other *Klebsiella* spp., GEN, CFX, CFV, AMC, CHL, FQs (ENR and PRA), SXT, and TET. The agents used to categorize staphylococci were GEN, OXA, AMP, AMC, cephalosporins (CFX and CFV), CHL, FQs (ENR and PRA), SXT, CLI, ERY, and TET. All isolates that showed resistance to OXA were considered insensitive to all tested β-lactams. Enterococci were categorized based on FQs (ENR and PRA), PEN, ERY, AMP, AMC, CHL, and TET. For streptococci, additionally, cephalosporins (CFX and CFV), CLI, OXA, and SXT were considered. For *Pseudomonas* spp., the MDR status was defined only based on non-susceptibility to FQs, CFV, and GEN, as they harbour numerous intrinsic resistances. NIT was excluded from the MDR definition since it achieves therapeutic concentrations in urine only [23].

### 4.6. Statistical Analysis

Statistical analyses were performed using the SAS 9.4 statistical software package. Descriptive statistics were performed for all data. To describe the data set, this was demonstrated for sex-specific parameters with two categories (e.g., male/female) using a binomial test and determination of 95% confidence limits. For sex-specific parameters with more than two categories (e.g., male/neutered/female/neutered), the chi-square test for equal distribution was used to determine whether these parameters were equally distributed in the data set. For comparison of categorical parameters between groups, a chi-square test was also performed. In cases where the chi-square test was not valid because the frequencies were too low, a Fisher exact test was performed. To answer the question of whether there were significant differences in AMR (categories I/S/R) between cats and dogs, exact chi-square tests were performed for each antimicrobial substance. For all AMR data at the ordinal level, the Wilcoxon–Mann–Whitney test was used to analyse whether there was a difference in AMR between cats and dogs for the individual bacteria and antimicrobial substances. If one of the species had only a single value in AMR, the other species was tested against that fixed value using a sign test. For all statistical analyses, the significance level was set at *p* < 0.05.

## 5. Conclusions

The results of this study revealed *E. coli*, followed by *Enterococcus* spp. and CoPS, as the most frequently isolated uropathogens in dogs and cats. Multidrug resistance to antimicrobial agents approved for the treatment of UTI in dogs and cats was demonstrated in 21.9% of all canine and in 24.5% of all feline isolates. The results highlight the importance of antimicrobial susceptibility testing prior to any antimicrobial treatment. The data obtained might also provide valuable information for the current use of antimicrobials. The application of antimicrobial substances should be well-considered and, whenever applicable, accompanied by continuously adjusted treatment guidelines to prevent the emergence of multidrug resistant uropathogens. Routine monitoring of AMR data is necessary to detect any unfavourable trends and to create awareness to the scientific community but also to inform veterinary practitioners about such trends in a timely manner. With regard to the putative risk of transmission of AMR/MDR uropathogens between humans and animals, extensive genome-wide comparative analyses would be beneficial to detect common clonal lineages, virulence gene profiles, AMR genes, and mobile genetic elements among isolates from these hosts.

## Figures and Tables

**Table 1:** Number of positive microbiological cultures, age, and sex of animals (1013 dogs and 536 cats).

		DOG		CAT	
		Sample Background	Positive Microbiological Cultures	Sample Background	Positive Microbiological Cultures
Submitted samples		1233	541 (43.9%) ^1^	629	242 (38.5%)
Average animal age ^2^		7.53 ± 4.36 years	8.39 ± 4.30 years	8.99 ± 4.98 years ^2^	10.03 ± 5.32 years
Age group	Sample size	1184		591	
	<2 years	149 (12.6%)	50 (9.6%)	41 (6.9%)	16 (7.3%)
	2–8 years	480 (40.6%)	185 (35.6%)	223 (37.7%)	66 (30.3%)
	>8 years	555 (46.9%)	285 (54.8%)	327 (55.3%)	137 (62.6%)
Sex ^3^	Sample size	1018		484	
	Male	52.9%	42.7%	60.7% ^4^	55.5%
	Female	47.1%	57.3%	39.3%	44.5%
Sex and neutering status ^5^	Sample size	419		130	
	Intact male	33.2% ^6^	17.7%	6.2%	2.1%
	Neutered Male	21.7%	24.1%	55.4% ^8^	43.8% ^8^
	Intact female	20.5%	22.7%	4.6%	6.3%
	Spayed Female	24.6%	35.5% ^7^	33.9% ^8^	47.9% ^8^

^1^ Dogs were 1.25 times more likely to develop a bacterial UTI than cats (odds ratio 1.2502); ^2^ the age of 49 dogs and 38 cats was not specified in the submission form; ^3^ the sex of 215 dogs and 145 cats was not provided in the submission form; ^4^ binomial test. There was a significant overrepresentation of samples from male cats in comparison to female cats (*p* < 0.0001); ^5^ for individuals that were presented at the University’s Clinic for Small Animals or for which the questionnaire was completed, additional data were available regarding the spay/neuter status; ^6^ chi-square test. The proportion of intact males was overrepresented (*p* = 0.0009); ^7^ chi-square test. The proportion of spayed females was overrepresented (*p* = 0.0234); ^8^ chi-square test. Cats were more likely to be neutered/spayed (*p* < 0.0001).

**Table 2:** Prevalence of bacterial species (697/313 isolates from dogs/cats) isolated from urinary tract samples, single vs. mixed infection with two, three, or more bacterial species.

Organism	Positive Microbiological Cultures (%)									
	Total		Mono-Infection		Mixed Infection					
					2 Bacterial Species		3 Bacterial Species		>3 Bacterial Species	
	Dog	Cat	Dog	Cat	Dog	Cat	Dog	Cat	Dog	Cat
*E. coli*	47.6	50.2	73.1 *	69.6 *	20.5	22.8	4.2	7.6	2.1	-
CoPS ^1^	14.8	4.1	72.8 *	61.5	12.8	30.8	6.4	7.7	2.1	-
*Enterococcus* spp.	9.9	16.2	37.5	41.2	40.7 *	43.1	27.1 *	15.7	10.2 *	-
*Proteus* spp.	9.1	3.5	58.7	27.3	19.0	54.5	12.7	18.2	9.5	-
*Sc. canis*	6.8	2.2	36.2	28.6	47.8	42.9	6.5	28.6	8.7	-
*Klebsiella* spp.	2.7	1.3	57.9	25.0	15.8	50.0	21.1	25.0	5.3	-
*EC*C ^2^	2.3	4.8	37.5	40.0	43.8	33.3	12.5	26.7	6.3	-
Other species **	2.2	5.5	20.0	62.5	33.3	37.5	13.3	-	33.3	-
*Pseudomonas* spp.	2.1	4.1	46.7	53.9	40.0	16.7	6.7	25.0	6.7	-
CoNS ^3^	1.4	7.9	70.0	60.0	35.7	32.0	-	8.0	7.1	-
*Corynebact. urealyticum*	0.4	-	66.7	-	33.3	-	-	-	-	-
*Pasteurella* spp.	0.3	1.6	100.0	40.0	-	40.0	-	20.0	-	-
*Acinetobacter* spp.	0.3	1.6	100.0	40.0	-	20.0	-	40.0	-	-

* Chi-square test. Bacterial species was significantly more often found in mono-infection or mixed infection (*p* < 0.05); ** other species included: *Aerococcus viridans*, *Alcaligenes faecalis, Citrobacter freundii, Corynebacterium* spp. other than *Corynebacterium urealyticum*, *Leclercia adecarboxylata*, *Morganella morganii*, *Providencia stuartii, Pseudescherichia vulneris*, *Rahnella* spp., *Raoultella planticola*, *Serratia marcescens*, and *Stenotrophomonas* spp.; ^1^ CoPS, coagulase-positive staphylococci; ^2^ *ECC*, *Enterobacter cloacae* complex; ^3^ CoNS, coagulase-negative staphylococci.

**Table 3:** Activity of various antimicrobials against *E. coli* isolates cultured from dogs with urinary tract infection.

	MIC Values (mg/L)														S	I	R	*n*	MIC_50_	MIC_90_
	0.00390625	0.0078125	0.015625	0.03125	0.0625	0.125	0.25	0.5	1	2	4	8	16	32	(%)	(%)	(%)		(mg/L)	(mg/L)
Amoxi/Clav *									14	174	86	41	6	11	94.9	-	5.1	332	2	8
Ampicillin *						1		4	24	163	59	6	75		77.4	-	22.6	332	2	≥16
Cephalexin *									1	2	120	167	24	18	94.6	-	5.4	332	8	16
Cefovecin *							116	167	32	6		11			96.7	-	3.3	332	0.5	1
Chloramphenicol **									3	14	200	95	3	17	94.9	-	5.1	332	4	8
Clindamycin											331				IR			331	≥4	≥4
Enrofloxacin *			146	112	11	20	10	4	2	2	24				91.5	1.2	7.3	331	0.03125	0.25
Erythromycin												331			IR			331	≥8	≥ 8
Gentamicin *						11	217	89	7	2		6			98.2	-	1.8	332	0.25	0.5
Oxacillin							1			1	330				IR			332	≥4	≥4
Penicillin G										1	3	328			IR			332	≥8	≥8
Pradofloxacin *		25	176	60	15	19	6	4	1	25					90.9	1.5	7.6	331	0.015625	0.25
Trim/Sulfa **							291	1	1	1	37				88.8	-	11.2	331	0.25	≥4
Tetracycline **								11	227	45	2	2	45		85.8	0.6	13.6	332	1	≥16

* Indicates that CLSI breakpoints derived from animal breakpoints were used. ** Indicates that CLSI breakpoints derived from human breakpoints were used. S = susceptible, R = resistant, I = intermediate, IR = intrinsic resistance. The dilution ranges tested are those contained in the white area, and values shown above this range are greater than or equal to the concentration shown. Values at the lower end of these ranges are less than or equal to the lowest concentration tested. Where available, breakpoints are indicated by a vertical line. Grey shaded areas indicate concentration of antimicrobial not tested. Amoxi/Clav, amoxicillin/clavulanic acid (2:1); Trim/Sulfa, trimethoprim-sulfamethoxazole (1:19). MICs were determined using standardized agar dilution methodology based upon the recommendation of the CLSI.

**Table 4:** Prevalence of bacterial isolates with a multidrug resistance pattern ^1^ from the total population, presented by bacterial and animal species.

	MDR Isolates Obtained from Dogs		MDR Isolates Obtained from Cats	
Bacterial Species/Group	No. of MDR/No. of Total	%	No. of MDR/No. of Total	%
*E. coli*	44/332	13.3	14/157	8.9
*Proteus* spp.	12/63	19.0	2/11	18.2
*EC*C *	4/16	25.0	4/15	26.7
*Klebsiella* spp.	4/19	21.1	1/4	25.0
*Enterococcus* spp.	30/67	44.8	29/50	58.0
CoPS **	41/104	39.4	9/13	69.2
*Sc. canis*	5/46	10.9	1/7	14.3
*S. felis*	-	-	0/22	0.0
*P. aeruginosa*	4/12	33.3	6/12	50.0
Total	144/659	21.9	66/291	24.5

^1^ According to Magiorakos et al. (2012), including XDR and PDR isolates [23]; * *EC*C, *Enterobacter cloacae* complex. ** CoPS, coagulase-positive staphylococci, bacterial species isolated from cats significantly more often revealed an MDR phenotype (*p* = 0.04, chi-square test).

**Table 5:** Activity of various antimicrobials against coagulase-positive staphylococci (CoPS) isolates cultured from dogs with urinary tract infection.

	MIC Values (mg/L)														S	I	R	*n*	MIC_50_	MIC_90_
	0.00390625	0.0078125	0.015625	0.03125	0.0625	0.125	0.25	0.5	1	2	4	8	16	32	(%)	(%)	(%)		(mg/L)	(mg/L)
Amoxi/Clav *					30	53	12	3	1		2		2		90.3	2.9	6.8	103	0.125	0.25
Ampicillin ^†^						35	15	18	8	9	3	5	8		49.5	17.8	32.7	101	0.5	≥8
Cephalexin ^‡^								49	41	6	2	1		3	94.1	-	5.9	102	1	2
Cefovecin ***							90	4	4	2	1	3			93.3	1	5.7	104	0.25	0.5
Chloramphenicol **									16	49	20	1	1	16	83.5	1	15.5	103	2	≥32
Clindamycin ^‡^				13	32	27	7	4	1	4	11				83.8	5.1	11.1	99	0.125	≥4
Enrofloxacin *			12	15	37	23	5	3	1		7				92.2	1	6.8	103	0.0625	0.5
Erythromycin **						59	16	6		3	1	18			78.6	3.9	17.5	103	0.125	≥8
Gentamicin **					10	70	11	1	1	2	2	4			96.0	3.9		101	0.125	0.25
Oxacillin **					36	58	4	2		1	3				94.2	-	5.8	104	0.125	0.125
Penicillin G **					23	14	5	4	8	6	8	34			36.3	-	63.7	102	1	≥8
Pradofloxacin *	10	5	9	32	34	5	2	1	2	4					93.3	3.8	2.9	104	0.03125	0.125
Trim/Sulfa **							74	20	1	2	7				93.3	-	6.7	104	0.25	0.5
Tetracycline **					26	29	7					6	28		64.6	6.3	29.2	96	0.125	≥16

* Indicates that CLSI breakpoints derived from animal breakpoints were used. ** Indicates that CLSI breakpoints derived from human breakpoints were used. *** Indicates that breakpoints from Fessler et al. (2017) were used [27]. ^†^ Indicates that CLSI breakpoints derived from the other animal species (referring to dog and cat) were used. ^‡^ Indicates that breakpoints from another body site (skin and soft tissue, SST) were used. S = susceptible, R = resistant, I = intermediate. The dilution ranges tested are those contained in the white area, and values shown above this range are greater than or equal to the concentration shown. Values at the lower end of these ranges are less than or equal to the lowest concentration tested. Where available, breakpoints are indicated by the vertical line (oxacillin: black line, *Staph. aureus*; grey line, all other CoPS). Grey shaded areas indicate concentration of antimicrobial not tested. Amoxi/Clav, amoxicillin/clavulanic acid (2:1); Trim/Sulfa, trimethoprim-sulfamethoxazole (1:19). MICs were determined using standardized agar dilution methodology based upon the recommendation of the CLSI. Differences between numbers in MIC values and susceptible/intermediate/resistant result from the validation due to oxacillin-resistant isolates.

**Table 6:** Activity of various antimicrobials against *E. coli* isolates cultured from cats with urinary tract infection.

	MIC Values (mg/L)														S	I	R	*n*	MIC_50_	MIC_90_
	0.00390625	0.0078125	0.015625	0.03125	0.0625	0.125	0.25	0.5	1	2	4	8	16	32	(%)	(%)	(%)		(mg/L)	(mg/L)
Amoxi/Clav *								1	12	82	34	19	3	6	94.3	-	5.7	157	2	8
Ampicillin ^†^								2	30	68	16	4	37		76.4	-	23.6	157	2	≥16
Cephalexin ^†^											92	53	7	5	96.8	-	3.2	157	4	8
Cefovecin *							87	56	6	1	1	5			96.2	0.6	3.2	156	0.25	0.5
Chloramphenicol **									3	2	94	51	3	4	97.5	-	2.5	157	4	8
Clindamycin											157				IR			157	≥4	≥4
Enrofloxacin ^†^			77	57	4	3	4			1	10				92.9	0.6	6.4	156	0.03125	0.125
Erythromycin										1		156			IR				>8	>8
Gentamicin ^†^						1	105	44	5		2				100.0	-	-		0.25	0.5
Oxacillin								1			156				IR				≥4	≥4
Penicillin G											2	155			IR				≥8	≥8
Pradofloxacin ^†^	2	12	95	26	4	6	1		2	9					93.0	1.3	5.7	157	0.015625	0.125
Trim/Sulfa **							138	5	1		13				91.7	-	8.3	157	0.25	0.5
Tetracycline **								15	111	11	1		19		87.9		12.1	157	1	≥16

* Indicates that CLSI breakpoints derived from animal breakpoints were used. ** Indicates that CLSI breakpoints derived from human breakpoints were used. ^†^ Indicates that CLSI breakpoints derived from the other animal species (referring to dog and cat) were used. S = susceptible, R = resistant, I = intermediate, IR = intrinsic resistance. The dilution ranges tested are those contained in the white area, and values shown above this range are greater than or equal to the concentration shown. Values at the lower end of these ranges are less than or equal to the lowest concentration tested. Where available, breakpoints are indicated by the vertical line. Grey shaded areas indicate concentration of antimicrobial not tested. Amoxi/Clav, amoxicillin/clavulanic acid (2:1); Trim/Sulfa, trimethoprim-sulfamethoxazole (1:19). MICs were determined using standardized agar dilution methodology based upon the recommendation of the CLSI.

**Table 7:** Activity of various antimicrobials against coagulase-positive staphylococci (CoPS) isolates cultured from cats with urinary tract infection.

	MIC Values (mg/L)														S	I	R	*n*	MIC_50_	MIC_90_
	0.00390625	0.0078125	0.015625	0.03125	0.0625	0.125	0.25	0.5	1	2	4	8	16	32	(%)	(%)	(%)		(mg/L)	(mg/L)
Amoxi/Clav *					1	2	2	3	2	1	1		1		38.5	15.4	46.2	13	0.5	4
Ampicillin *						2		2	1			1	7		15.4	15.4	69.2	13	≥16	≥16
Cephalexin ^†‡^								3	2	2	2	2		2	53.8	-	46.2	13	2	≥32
Cefovecin ***							3	2	2	3		2			75.0	-	25.0	12	1	8
Chloramphenicol **									1	5	6			1	92.3	-	7.7	13	4	4
Clindamycin ^†‡^					1	5		1			6				53.8	-	46.2	13	0.5	≥4
Enrofloxacin ^†^				1	2	3					7				46.2	-	53.8	13	≥4	≥4
Erythromycin **						2	3	1				7			46.2	-	53.8	13	≥8	≥8
Gentamicin **						3	3				2	4			66.7	33.3		12	4	≥8
Oxacillin **					3	5	2	1		1	1				76.9	-	23.1	13	0.125	2
Penicillin G **					2		1	1				9			15.4	-	84.6	13	≥8	≥8
Pradofloxacin ^†^			1		4	1		2	2	3					46.2	30.8	23.1	13	0.5	≥2
Trim/Sulfa **							7				6				53.8	-	46.2	13	0.25	≥4
Tetracycline **					1	2	3						7		46.2	-	53.8	13	≥16	≥16

* Indicates that CLSI breakpoints derived from animal breakpoints were used. ** Indicates that CLSI breakpoints derived from human breakpoints were used. *** Indicates that breakpoints from Fessler et al. (2017) were used [27]. ^†^ Indicates that CLSI breakpoints derived from the other animal species (referring to dog and cat) were used. ^‡^ Indicates that breakpoints from another body site (skin and soft tissue, SST) were used. S = susceptible, R = resistant, I = intermediate. The dilution ranges tested are those contained in the white area, and values shown above this range are greater than or equal to the concentration shown. Values at the lower end of these ranges are less than or equal to the lowest concentration tested. Where available, breakpoints are indicated by the vertical line (oxacillin: black line, *Staph. aureus*; grey line, all other CoPS). Grey shaded areas indicate concentration of antimicrobial not tested. Amoxi/Clav, amoxicillin/clavulanic acid (2:1); Trim/Sulfa, trimethoprim-sulfamethoxazole (1:19). MICs were determined using standardized agar dilution methodology based upon the recommendation of the CLSI. Differences between numbers in MIC values and susceptible/intermediate/resistant result from the validation due to oxacillin-resistant isolates.

**Table 8:** Distribution of clinical resistance of 559 uropathogenic bacterial isolates from UTI samples in dogs (*n* = 301) and cats (*n* = 139) to nitrofurantoin.

	All Bacterial Isolates				MDR ^1^ Bacteria			
	DOG		CAT		DOG		CAT	
	S (%)	*n* *	S (%)	*n* *	S (%)	*n* **	S (%)	*n* **
*E. coli*	92.5	214	91.9	111	90.0	30	9.5	10
*Klebsiella* spp.	50.0	12	50.0	4	0.0	3	0.0	1
*EC*C ^2^	28.6	7	41.7	12	0.0	1	25.0	4
CoPS ^3^	100.0	79	100.0	7	100.0	30	100.0	4
*Enterococcus* spp.	90.9	44	83.8	37	94.4	18	77.3	22
*Sc. canis*	100.0	27	100.0	5	100.0	3	100.0	1

Breakpoints and interpretation as recommended by the CLSI [29]; (S) susceptible ≥ 17 mm, (I) intermediate 15–16 mm, (R) resistant ≤ 14 mm; ^1^ according to Magiorakos et al. (2012) [23]; * all isolates tested for NIT resistance; ** all MDR isolates tested for NIT resistance. ^2^ *EC*C, *Enterobacter cloacae* complex; ^3^ CoPS, coagulase-positive staphylococci.

**Table 9:** Characteristics of third-generation cephalosporin-resistant *Enterobacterales* from dogs and cats.

Bacterial Species	Animal	Sample	Sample Origin	Strain ID	Cefovecin(Merlin Micronaut-S)	ESBL-Alert(VITEK^®^2)
*E. coli*	Dog	Urine	Hospital 3	IHIT42968	R (>4)	Negative
*E. coli*	Dog	Urine	Practice 1	n.s.	R (>4)	-
*E. coli*	Dog	Urolith	Hospital 2	IHIT43802	R (>4)	Phenotypic ESBL
*E. coli*	Dog	Urine	Hospital 2	IHIT41651	R (>4)	Phenotypic ESBL
*E. coli*	Dog	Urine	Hospital 1	IHIT41754	R (>4)	Negative
*E. coli*	Dog	Urine	Practice 24	IHIT42516	R (>4)	Phenotypic ESBL
*E. coli*	Dog	Urine	Hospital 2	n.s.	R (>4)	-
*E. coli*	Dog	Urine	Practice 1	n.s.	R (>4)	-
*E. coli* ^1^	Dog	Urine	Hospital 1	IHIT43540	R (>4)	Phenotypic ESBL
*E. coli* ^1^	Dog	Urine	Hospital 1	IHIT43640	R (>4)	Phenotypic ESBL
*E. coli* ^1^	Dog	Urine	Hospital 1	IHIT43899	R (>4)	Phenotypic ESBL
*E. coli*	Cat	Urine	Practice 2	IHIT42192	R (>4)	Phenotypic ESBL
*E. coli*	Cat	Urine	Practice 2	n.s.	R (>4)	-
*E. coli* hemolytic	Cat	Urine	Hospital 3	IHIT42807	R (>4)	Negative
*E. coli* hemolytic	Cat	Urine	Hospital 1	IHIT43661	R (>4)	Negative
*E. coli* hemolytic	Cat	Urine	Hospital 3	n.s.	R (>4)	-
*EC*C	Dog	Urine	Hospital 2	IHIT43208	R (>4)	Phenotypic ESBL
*EC*C	Dog	Urine	Hospital 1	IHIT41647	R (>4)	Phenotypic ESBL
*EC*C	Dog	Urine	Practice 3	IHIT42531	R (>4)	Phenotypic ESBL
*EC*C	Cat	Urine	Practice 5	IHIT42547	R (>4)	Phenotypic ESBL
*EC*C	Cat	Bladder swab	Hospital 3	IHIT42613	R (>4)	Phenotypic ESBL
*EC*C	Cat	Urine	Hospital 2	IHIT42964	R (>4)	Phenotypic ESBL
*K. pneumoniae*	Dog	Urine	Hospital 2	IHIT41655	R (>4)	Negative
*P. mirabilis*	Dog	Urine	Hospital 4	IHIT41742	R (>4)	Negative
*P. mirabilis*	Dog	Urine	Practice 1	IHIT43910	R (>4)	Phenotypic ESBL

n.s.: not stored and therefore not available for further analysis; ^1^ isolate derived from same dog obtained from different samples at different times; *EC*C, *Enterobacter cloacae* complex.

**Table 10:** Most common bacterial uropathogens accounting for 93% of all canine and feline UTIs according to J. A. Barsanti [19].

Gram-Negative Organisms	Gram-Positive Organisms
*E. coli*	*Staphylococcus* sp. *
*Proteus* sp.	*Streptococcus* sp.
*Klebsiella* sp.	*Enterococcus* sp.
*Pseudomonas* sp.	
*Enterobacter* sp.	

* In cats, the most common staphylococcal species is *S. felis*.

## Data Availability

All data are presented in the text and tables.

## Supplementary Materials

The following are available online at https://www.mdpi.com/article/10.3390/antibiotics11121730/s1, Figure S1: Questionnaire created for the project to collect additional information regarding signalment (age, sex, castration status), clinical symptoms and suspected diagnosis, type of sample collection, and previous treatment; Table S1: Distribution of comorbidities among dogs and cats with symptoms of UTI; Table S2: Activity of various antimicrobials against 63 *Proteus* spp. isolates cultured from dogs with urinary tract infection; Table S3: Activity of various antimicrobials against 16 *Enterobacter cloacae* complex (ECC) isolates cultured from dogs with urinary tract infection; Table S4: Activity of various antimicrobials against 19 *Klebsiella* spp. isolates cultured from dogs with urinary tract infection; Table S5: Activity of various antimicrobials against 15 *Pseudomonas aeruginosa* isolates cultured from dogs with urinary tract infection; Table S6: Activity of various antimicrobials against 66 enterococci isolates cultured from dogs with urinary tract infection; Table S7: Activity of various antimicrobials against 46 *Sc. canis* isolates cultured from dogs with urinary tract infection; Table S8: Activity of various antimicrobials against 11 *Proteus* spp. isolates cultured from cats with urinary tract infection; Table S9: Activity of various antimicrobials against 15 *Enterobacter cloacae* complex (ECC) isolates cultured from cats with urinary tract infection; Table S10: Activity of various antimicrobials against 13 *Pseudomonas aeruginosa* isolates cultured from cats with urinary tract infection; Table S11: Activity of various antimicrobials against 50 enterococci isolates cultured from cats with urinary tract infection; Table S12: Activity of various antimicrobials against coagulase-negative staphylococci (CoNS) isolates cultured from cats with urinary tract infection; Table S13: Number of susceptible isolates among cats and dogs suffering of UTI presented by animal and antibiotic.
