# Supplementary material for: Prevalence and Antimicrobial Resistance of Bacterial Uropathogens Isolated from Dogs and Cats
Source: Antibiotics (Basel). 2022 Dec 1;11(12):1730. doi: 10.3390/antibiotics11121730 (PMC9774110; doi:10.3390/antibiotics11121730)
Supplement: Supplementary file 1 [file antibiotics-11-01730-s001.zip › antibiotics-2042814-supplementary.pdf]

JUSTUS-LIEBIG-UNIVERSITÄT GIESSEN

FACHBEREICH 10
Veterinärmedizin

Sophie Aurich / Prof. Dr. Christa Ewers
Institut für Hygiene und Infektionskrankheiten der Tiere
Justus-Liebig-Universität Gießen
Frankfurter Str. 85-89
35392 Gießen

Fax: 0641 / 99 383 09
Telefon: 0641 / 99 383 11

Projekt: *„Keimspektrum und Resistenzlage bei bakteriellen Harnwegsinfektionen"*

Name und Alter des Tieres: ....

☐ **Hund**		☐ **Katze**	
		☐ Freigänger	☐ Wohnungskatze
☐ männlich	☐ weiblich	☐ Kastriert	
☐ weitere Tiere im Haushalt:			

Grund der Vorstellung

☐ **Urogenitalinfektion**			
☐ akut	☐ chronisch	☐ rezidivierend	☐ Nachkontrolle
☐ **andere Erkrankung(en):**			
☐ akut	☐ chronisch	☐ rezidivierend	☐ Nachkontrolle
☐ **Vorerkrankungen:**			

Symptome

☐ Pollakisurie	☐ Strangurie	☐ Dysurie	☐ Hämaturie
☐ Schmerzhaftigkeit:	☐ geringgradig	☐ mittelgradig	☐ hochgradig

Probenentnahme

☐ Zystozentese	☐ Spontanurin	☐ Katheter

Vorbehandlung

☐ Amoxicillin/ Clavulansäure	☐ Trimethoprim/ Sulfamethoxazol	☐ Trimethoprim/ Sulfadoxin	☐ Trimethoprim/ Sulfadiazin
☐ Amoxicillin	☐ Cephalexin	☐ Spectinomycin	☐ Enrofloxacin
☐ Penicillin G	☐ Cefovecin	☐ Gentamicin	☐ Pradofloxacin
☐ Ampicillin	☐ Doxycyclin	☐ Lincomycin	☐ Marbofloxacin
☐ Metronidazol	☐ andere:		☐ **keine**
Zeitpunkt/Dauer:			

☐ Einsatz einer Vakzine (tierspezifisch) aufgrund einer Harnwegsinfektion<br>Erreger/Datum:	☐ Katheterisiert

Figure S1. Questionnaire created for the project to collect additional information regarding signalment (age, sex, castration status), clinical symptoms and suspected diagnosis, type of sample collection, and previous treatment.

Table S1. Distribution of comorbidities among 113 dogs and 48 cats with symptoms of UTI

**Comorbidities**	**Dog**	**Cat**	**Total**
	**n (%)**	**n (%)**	**n (%)**
**None**	**63 (50.0)**	**17 (34.0)**	**80 (45.5)**
Urolithiasis	12 (9.5)	12 (24.0)	24 (13.6)
Renal disease	8 (6.3)	6 (12.0)	14 (8.0)
Disorders of micturition	7 (5.6)	1 (2.0)	8 (4.5)
Hepatic disease	6 (4.8)	1 (2.0)	7(4.0)
Diabetes mellitus	1 (0.8)	5 (10.0)	6 (3.4)
Cardiovascular disease	3 (2.4)	3 (6.0)	6 (3.4)
other [a]	4 (3.2)	1 (2.0)	5 (2.8)
Hepatic disease	4 (3.2)	1 (2.0)	5 (2.8)
TCC [b]	4 (3.2)	-	4 (2.3)
Hyperadrenocorticism	4 (3.2)	-	4 (2.3)
Anemia	3 (2.4)	-	3 (1.7)
Hyperthyroidism	-	3 (6.0)	3 (1.7)
Hypothyroidism	3 (42.4)	-	3 (1.7)
Other endocrine diseases	2 (1.6)	-	2 (1.1)
Urethrorectal fistula	2 (1.6)	-	2 (1.1)
Bladder polyp	2 (1.6)		2 (1.1)
Neoplasia outside UT[c]	2 (1.6)	-	2 (2.1)
Perineal urethrostomy	-	1 (2.0)	1 (0.6)
**Total**	**126 (100)**	**50 (100)**	**176 (100)**

Total number of comorbidities exceeds number of animals because some had multiple comorbidities reported. [a] Feline bronchial asthma, anal sac infection, otitis, skin mass; [b] Transitional Cell Carcinoma; [c] urinary tract; the total number of comorbidities exceeds the number of animals because some had multiple comorbidities listed.

Table S2. Activity of various antimicrobials against 63 *Proteus* spp. isolates cultured from dogs with urinary tract infection

	MIC values (mg/L)														S	I	R	n	MIC_{50}	MIC_{90}
	0.00390625	0.0078125	0.015625	0.03125	0.0625	0.125	0.25	0.5	1	2	4	8	16	32	(%)	(%)	(%)		(mg/L)	(mg/L)
Amoxi/Clav•							3	20	21	5	5	7		2	96.8	-	3.2	63	1	8
Ampicillin•							3	7	32	1	1		19		69.8	-	30.2	63	1	≥ 16
Cephalexin*											1	23	33	6	90.5	-	9.5	63	16	16
Cefovecin*							60	1				2			96.8	-	3.2	63	0.25	0.25
Chloramphenicol**									1	1	13	34	5	9	85.7	0.0	14.3	63	8	≥ 32
Clindamycin											63					IR		63	≥ 4	≥ 4
Enrofloxacin*					5	36	6	2	1	1	12				77.8	3.2	19.0	61	0.125	≥ 4
Erythromycin											1	62				IR		63	≥ 8	≥ 8
Gentamicin*						1	9	44	1	4	3	1			98.4	-	1.6	63	0.5	2
Oxacillin											63	0				IR		63	≥ 4	≥ 4
Penicillin G							1		3	19	17	23				IR		63	4	≥ 8
Pradofloxacin•				1		38	7	2	3	12					73.0	7.9	19.0	63	0.125	≥ 2
Trim/Sulfa**							46	2		1	14				77.8	-	22.2	63	0.25	≥ 4
Tetracycline												4	59			IR		63	≥ 16	≥ 16

* Indicates that CLSI breakpoints derived from animal breakpoints were used. ** Indicates that CLSI breakpoints derived from human breakpoints were used.• Indicates that CLSI breakpoints derived from *E. coli* were used. S=susceptible, R=resistant, I= intermediate , IR=intrinsic resistance. The dilution ranges tested are those contained in the white area, and values shown above this range are greater than or equal to the concentration shown. Values at the lower end of these ranges are less than or equal to the lowest concentration tested. Where available, breakpoints are indicated by a vertical line. Grey shaded areas indicate concentration of antimicrobial not tested. Amoxi/Clav, amoxicillin/clavulanic acid (2:1); Trim/Sulfa, trimethoprim-sulfamethoxazole (1:19). MICs were determined using standardized agar dilution methodology based upon the recommendation of the CLSI.

Table S3. Activity of various antimicrobials against 16 *Enterobacter cloacae* complex (*ECC*) isolates cultured from dogs with urinary tract infection

	MIC values (mg/L)														S	I	R	n	MIC_{50}	MIC_{90}
	0.00390625	0.0078125	0.015625	0.03125	0.0625	0.125	0.25	0.5	1	2	4	8	16	32	(%)	(%)	(%)		(mg/L)	(mg/L)
Amox/clav														16		IR		16	≥ 32	≥ 32
Ampicillin											1	1	14			IR		16	≥ 16	≥ 16
Cephalexin													1	15		IR		16	≥ 32	≥ 32
Cefovecin•								1	7	2	3	3			62.5	18.8	18.8	16	1	8
Chloramphenicol**											6	6	1	3	81.3	-	18.8	16	8	≥ 32
Clindamycin											16					IR		16	≥ 4	≥ 4
Enrofloxacin*			1	9			1	2	1		2				81.3	6.3	12.5	16	0.03125	4
Erythromycin												16				IR		16	≥ 8	≥ 8
Gentamicin*							12	1	1			2			87.5	-	12.5	16	0.25	8
Oxacillin											16					IR		16	≥ 4	≥ 4
Penicillin G												16				IR		16	≥ 8	≥ 8
Pradofloxacin•	1		1	8		1	1	2	1	1					75.0	18.8	6.25	16	0.03125	1
Trim/Sulfa**							12	2			2				87.5	-	12.5	16	0.25	≥ 4
Tetracycline**								11	227	45	2	2	45		85.8	0.6	13.6	16	1	≥ 16

* Indicates that CLSI breakpoints derived from animal breakpoints were used. ** Indicates that CLSI breakpoints derived from human breakpoints were used. • Indicates that CLSI breakpoints derived from *E. coli* were used. S=susceptible, R=resistant, I= intermediate, IR=intrinsic resistance. The dilution ranges tested are those contained in the white area, and values shown above this range are greater than or equal to the concentration shown. Values at the lower end of these ranges are less than or equal to the lowest concentration tested. Where available, breakpoints are indicated by a vertical line. Grey shaded areas indicate concentration of antimicrobial not tested. Amoxi/Clav, amoxicillin/clavulanic acid (2:1); Trim/Sulfa, trimethoprim-sulfamethoxazole (1:19). MICs were determined using standardized agar dilution methodology based upon the recommendation of the CLSI.

Table S4. Activity of various antimicrobials against 19 *Klebsiella* spp. isolates cultured from dogs with urinary tract infection

	MIC values (mg/L)														S	I	R	n	MIC_{50}	MIC_{90}
	0.00390625	0.0078125	0.015625	0.03125	0.0625	0.125	0.25	0.5	1	2	4	8	16	32	(%)	(%)	(%)		(mg/L)	(mg/L)
Amoxi/Clav•									7	6	2			4	78.9	-	21.1	19	2	≥ 32
Ampicillin												2	17			IR		19	≥16	≥16
Cephalexin*											13	2		4	63.2	-	36.8	19	4	≥ 32
Cefovecin•							10	4		3	1	1			89.5	5.3	5.3	19	0.25	4
Chloramphenicol**										3	12		2	2	89.5	-	10.5	19	4	≥ 32
Clindamycin											19					IR		19	≥ 4	≥ 4
Enrofloxacin*				13	1	1	1			1	2				84.2	5.3	10.5	19	0.03125	≥ 4
Erythromycin												19				IR		19	≥ 8	≥ 8
Gentamicin*						1	16	2							100.0	-	-	19	0.25	0.5
Oxacillin											19					IR		19	≥ 4	≥ 4
Penicillin G												19				IR		19	≥ 8	≥ 8
Pradofloxacin•				13	1	1		1	1	2					78.9	10.5	10.5	19	0.03125	≥ 2
Trim/Sulfa**							19								100.0	-	-	19	0.25	0.25
Tetracycline**								3	10	1	2	2	1		84.2	10.5	5.3	19	1	8

* Indicates that CLSI breakpoints derived from animal breakpoints were used. ** Indicates that CLSI breakpoints derived from human breakpoints were used. • Indicates that CLSI breakpoints derived from *E. coli* were used. S=susceptible, R=resistant, I= intermediate, IR=intrinsic resistance. The dilution ranges tested are those contained in the white area, and values shown above this range are greater than or equal to the concentration shown. Values at the lower end of these ranges are less than or equal to the lowest concentration tested. Where available, breakpoints are indicated by a vertical line (black line *Klebsiella pneumoniae*, grey line all other *Klebsiella* spp.). Grey shaded areas indicate concentration of antimicrobial not tested. Amoxi/Clav, amoxicillin/clavulanic acid (2:1); Trim/Sulfa, trimethoprim-sulfamethoxazole (1:19). MICs were determined using standardized agar dilution methodology based upon the recommendation of the CLSI.

Table S5. Activity of various antimicrobials against 12 *Pseudomonas aeruginosa* isolates cultured from dogs with urinary tract infection

	**MIC values (mg/L)**														**S**	**I**	**R**	**n**	**MIC50**	**MIC90**
	0.00390625	0.0078125	0.015625	0.03125	0.0625	0.125	0.25	0.5	1	2	4	8	16	32	(%)	(%)	(%)		(mg/L)	(mg/L)
Amox/Clav														12		IR		12	≥ 32	≥ 32
Ampicillin													12			IR		12	≥ 16	≥ 16
Cephalexin														12		IR		12	≥ 32	≥ 32
Cefovecin***												12			-	-	100.0	12	≥ 4	≥ 4
Chloramphenicol													4	8		IR		12	≥ 32	≥ 32
Clindamycin											12					IR		12	≥ 4	≥ 4
Enrofloxacin†‡							1	7	1		3				66.7	8.3	25.0	12	0.5	≥ 4
Erythromycin												12				IR		12	≥ 8	≥ 8
Gentamicin*								4	5	1	1	1			83.3	8.3	8.3	12	1	4
Oxacillin											12					IR		12	≥ 4	≥ 4
Penicillin G												12				IR		12	≥ 8	≥ 8
Pradofloxacin							2	7	1	2					16.6	66.7	16.6	12	0.5	≥ 2
Trim/Sulfa									1	4	7					IR		12	≥ 4	≥ 4
Tetracycline													12			IR		12	≥ 16	≥ 16

* Indicates that CLSI breakpoints derived from animal breakpoints were used. *** Indicates that breakpoints from Fessler et al. (2017) were used [27]. † Indicates that CLSI breakpoints derived from the other animal species (referring to dog and cat) were used. ‡ Indicates that breakpoints from another body site (skin and soft tissue, SST) were used. S=susceptible, R=resistant, I= intermediate, IR=intrinsic resistance. The dilution ranges tested are those contained in the white area, and values shown above this range are greater than or equal to the concentration shown. Values at the lower end of these ranges are less than or equal to the lowest concentration tested. Where available, breakpoints are indicated by a vertical line. Grey shaded areas indicate concentration of antimicrobial not tested. Amoxi/Clav, amoxicillin/clavulanic acid (2:1); Trim/Sulfa, trimethoprim-sulfamethoxazole (1:19). MICs were determined using standardized agar dilution methodology based upon the recommendation of the CLSI.

Table S6. Activity of various antimicrobials against 66 enterococci isolates cultured from dogs with urinary tract infection

	MIC values (mg/L)														S	I	R	n	MIC_{50}	MIC_{90}
	0.00390625	0.0078125	0.015625	0.03125	0.0625	0.125	0.25	0.5	1	2	4	8	16	32	(%)	(%)	(%)		(mg/L)	(mg/L)
Amoxi/Clav					2	1	11	37	4	2		3		4	93.8	-	6.3	64	0.5	8
Ampicillin**						2	5	23	28	1		2	5		92.4	-	7.6	66	1	8
Cephalexin										1		3	5	57		IR		66	≥ 32	≥ 32
Cefovecin										2	6	58				IR		66	≥ 8	≥ 8
Chloramphenicol**									2	8	44	5	4	2	96.9	-	3.1	65	4	8
Clindamycin						1			1	1	63					IR		66	≥ 4	≥ 4
Enrofloxacin						2	19	33	3	3	6				81.8	9.1	9.1	66	0.5	2
Erythromycin**						6	3	4	8	15	16	14			19.7	59.1	21.2	66	2	≥ 8
Gentamicin									1	7	15	43				IR		66	≥ 8	≥ 8
Oxacillin										1	65				2.5	-	97.5	66	≥ 4	≥ 4
Penicillin G**						2	2	4	15	33	2	8			100.0	-	-	66	2	≥ 8
Pradofloxacin					2	18	33	4	1	8					80.3	7.6	12.1	66	0.25	≥ 2
Trim/Sulfa							9		1		56					IR		66	≥ 4	≥ 4
Tetracycline**						1	2	7	24	2	2	1	27		57.6	1.5	40.9	66	1	≥ 16

** Indicates that CLSI breakpoints derived from human breakpoints were used. S=susceptible, R=resistant, I= intermediate, IR=intrinsic resistance. The dilution ranges tested are those contained in the white area, and values shown above this range are greater than or equal to the concentration shown. Values at the lower end of these ranges are less than or equal to the lowest concentration tested. Where available, breakpoints are indicated by a vertical line. Grey shaded areas indicate concentration of antimicrobial not tested. Amoxi/Clav, amoxicillin/clavulanic acid (2:1); Trim/Sulfa, trimethoprim-sulfamethoxazole (1:19). MICs were determined using standardized agar dilution methodology based upon the recommendation of the CLSI.

Table S7. Activity of various antimicrobials against 46 *Sc. canis* isolates cultured from dogs with urinary tract infection

	MIC values (mg/L)														S	I	R	n	MIC_{50}	MIC_{90}
	0.00390625	0.0078125	0.015625	0.03125	0.0625	0.125	0.25	0.5	1	2	4	8	16	32	(%)	(%)	(%)		(mg/L)	(mg/L)
Amoxi/Clav†					44	1									100.0	-	-	45	0.0625	0.0625
Ampicillin†						45		1							97.8	2.2	-	46	0.125	0.125
Cephalexin								43	1						100.0	-	-	44	0.5	0.5
Cefovecin***							45	1							100.0	-	-	46	0.25	0.25
Chloramphenicol**									17	27	1	1			97.8	2.2	-	46	2	2
Clindamycin**				1	12	22	7	1			3				91.3	2.2	6.5	46	0.125	0.5
Enrofloxacin*							13	28	3	1					91.1	8.9	-	45	0.5	0.5
Erythromycin**						39		2			1	3			86.7	4.4	8.9	45	0.125	0.5
Gentamicin										3	34	8				LLR		45	4	≥ 8
Oxacillin					38	2		1	1	1					93.0	-	7.0	43	0.0625	0.125
Penicillin G**					45										100.0	-	-	45	0.0625	0.0625
Pradofloxacin					7	33	1	3	1						91.1	8.9	-	45	0.125	0.25
Trim/Sulfa**							43	2		1					97.8	2.2	-	46	0.25	0.25
Tetracycline						1	4	11	15	5	1		7		70.5	11.4	18.8	44	1	≥ 16

* Indicates that CLSI breakpoints derived from animal breakpoints were used. ** Indicates that CLSI breakpoints derived from human breakpoints were used. *** Indicates that breakpoints from Fessler et al. (2017) were used [27]. † Indicates that CLSI breakpoints derived from the other animal species (referring to dog and cat) were used. S=susceptible, R=resistant, I= intermediate, LLR=Low-level resistance to aminoglycosides. The dilution ranges tested are those contained in the white area, and values shown above this range are greater than or equal to the concentration shown. Values at the lower end of these ranges are less than or equal to the lowest concentration tested. Where available, breakpoints are indicated by a vertical line. Grey shaded areas indicate concentration of antimicrobial not tested. Amoxi/Clav, amoxicillin/clavulanic acid (2:1); Trim/Sulfa, trimethoprim-sulfamethoxazole (1:19).MICs were determined using standardized agar dilution methodology based upon the recommendation of the CLSI.

Table S8. Activity of various antimicrobials against 11 *Proteus* spp. isolates cultured from cats with urinary tract infection

	**MIC values (mg/L)**														**S**	**I**	**R**	**n**	**MIC_{50}**	**MIC_{90}**
	0.00390625	0.0078125	0.015625	0.03125	0.0625	0.125	0.25	0.5	1	2	4	8	16	32	(%)	(%)	(%)		(mg/L)	(mg/L)
Amoxi/Clav•								6	2	1	1	1			100	-	-	11	0.5	4
Ampicillin**								1	7		1		2		81.8	18.2[1]		11	1	≥ 16
Cephalexin†											1	4	5	1	90.9	-	9.1	11	≥ 16	≥ 16
Cefovecin†							9	1			1				90.9	9.1	-	11	0.25	0.5
Chloramphenicol*											3	6		2	81.8	-	18.2	11	8	≥ 32
Clindamycin											11					IR		11	≥ 4	≥ 4
Enrofloxacin†					1	7	2				1				90.9	-	9.1	11	0.125	0.25
Erythromycin												11				IR		11	≥ 8	≥ 8
Gentamicin†							1	8	1			1			90.9	-	9.1	11	0.5	1
Oxacillin											11					IR		11	≥ 4	v 4
Penicillin G										7	1	3				IR		11	2	≥ 8
Pradofloxacin†					1	8	1			1					90.9	-	9.1	11	0.125	0.25
Trim/Sulfa*							8		1		2				81.8	-	18.2	11	0.25	≥ 4
Tetracycline									1				10			IR		11	≥ 16	≥ 16

* Indicates that CLSI breakpoints derived from human breakpoints were used. † Indicates that CLSI breakpoints derived from the other animal species (referring to dog and cat) were used. • Indicates that CLSI breakpoints derived from *E. coli* were used. S=susceptible, R=resistant, I= intermediate, IR=intrinsic resistance. The dilution ranges tested are those contained in the white area, and values shown above this range are greater than or equal to the concentration shown. Values at the lower end of these ranges are less than or equal to the lowest concentration tested. Where available, breakpoints are indicated by a vertical line. Grey shaded areas indicate concentration of antimicrobial not tested. Amoxi/Clav, amoxicillin/clavulanic acid (2:1); Trim/Sulfa, trimethoprim-sulfamethoxazole (1:19). MICs were determined using standardized agar dilution methodology based upon the recommendation of the CLSI. [1]For *Proteus* spp. in combination with AMP, differentiation between intermediate and resistant isolates was not possible due to the test design of the layout used in our study. The concentration ranged between 0,125 to 8 mg/L, while the CLSI breakpoint [52] designates 16 mg/L as intermediate and equal or greater to 32 mg/L as resistant. In this case, only the classification into susceptible (≤8 mg/L) and non-susceptible was performed.

Table S9. Activity of various antimicrobials against 15 *Enterobacter cloacae* complex (*ECC*) isolates cultured from cats with urinary tract infection

	**MIC values (mg/L)**														**S**	**I**	**R**	**n**	**MIC50**	**MIC90**
	0.00390625	0.0078125	0.015625	0.03125	0.0625	0.125	0.25	0.5	1	2	4	8	16	32	(%)	(%)	(%)		(mg/L)	(mg/L)
Amox/Clav								1					2	12		IR		15	≥ 32	≥ 32
Ampicillin							1				1	2	11			IR		15	≥ 16	≥ 16
Cephalexin										1		1	1	12		IR		15	≥ 32	≥ 32
Cefovecin•							1		4	6	1	| 3			73.3	6.7	20.0	15	2	8
Chloramphenicol*										1	4	8		| 2	86.7	-	13.3	15	8	≥ 32
Clindamycin											15					IR		15	≥ 4	≥ 4
Enrofloxacin†			2	5	2	1		1			| 4				73.3	-	26.7	15	0.0625	≥ 4
Erythromycin												15				IR		15	≥ 8	≥ 8
Gentamicin†							12			1		| 2			86.7	-	13.3	15	0.25	≥ 8
Oxacillin											15					IR		15	≥ 4	≥ 4
Penicillin G									1			14				IR		15	≥ 8	≥ 8
Pradofloxacin†	1		2	3	2	1	1	1		| 4					86.7	-	13.3	15	0.0625	≥ 2
Trim/Sulfa*							12				| 3				80.0	-	20.0	15	0.25	≥ 4
Tetracycline*								1	3	6	2	1	| 2		80.0	6.7	13.3	15	2	≥ 16

* Indicates that CLSI breakpoints derived from human breakpoints were used. • Indicates that CLSI breakpoints derived from *E. coli* were used. † Indicates that CLSI breakpoints derived from the other animal species (referring to dog and cat) were used. S=susceptible, R=resistant, I= intermediate, IR=intrinsic resistance. The dilution ranges tested are those contained in the white area, and values shown above this range are greater than or equal to the concentration shown. Values at the lower end of these ranges are less than or equal to the lowest concentration tested. Where available, breakpoints are indicated by a vertical line. Grey shaded areas indicate concentration of antimicrobial not tested. Amoxi/Clav, amoxicillin/clavulanic acid (2:1); Trim/Sulfa, trimethoprim-sulfamethoxazole (1:19). MICs were determined using standardized agar dilution methodology as recommended by the CLSI.

Table S10. Activity of various antimicrobials against 12 *Pseudomonas aeruginosa* isolates cultured from cats with urinary tract infection

	MIC values (mg/L)														S	I	R	n	MIC_{50}	MIC_{90}
	0.00390625	0.0078125	0.015625	0.03125	0.0625	0.125	0.25	0.5	1	2	4	8	16	32	(%)	(%)	(%)		(mg/L)	(mg/L)
Amox/Clav														12		IR		12	≥ 32	≥ 32
Ampicillin													12			IR		12	≥ 16	≥ 16
Cephalexin														12		IR		12	≥ 32	≥ 32
Cefovecin*												12			-	-	100.0	12	≥ 4	≥ 4
Chloramphenicol													3	9		IR		12	≥ 32	≥ 32
Clindamycin											12					IR		12	≥ 4	≥ 4
Enrofloxacin‡							6		1	2	3				50.0	25.0	25.0	12	1	≥ 4
Erythromycin												12				IR		12	≥ 12	≥ 12
Gentamicin†								5	4	1	2				83.3	16.6	-	12	1	4
Oxacillin					1						11					IR		12	≥ 4	≥ 4
Penicillin G												12				IR		12	≥ 8	≥ 8
Pradofloxacin						1	5		2	4					50.0	16.6	33.3	12	1	≥ 2
Trim/Sulfa										4	8					IR		12	≥ 4	≥ 4
Tetracycline												1	11			IR		12	≥ 16	≥ 16

* Indicates that breakpoints from Fessler et al. (2017) were used[27] . † Indicates that CLSI breakpoints derived from the other animal species (referring to dog and cat) were used. ‡ Indicates that breakpoints from another body site (skin and soft tissue, SST) were used. S=susceptible, R=resistant, I= intermediate, IR=intrinsic resistance. The dilution ranges tested are those contained in the white area, and values shown above this range are greater than or equal to the concentration shown. Values at the lower end of these ranges are less than or equal to the lowest concentration tested. Where available, breakpoints are indicated by a vertical line. Grey shaded areas indicate concentration of antimicrobial not tested. Amoxi/Clav, amoxicillin/clavulanic acid (2:1); Trim/Sulfa, trimethoprim-sulfamethoxazole (1:19). MICs were determined using standardized agar dilution methodology based upon the recommendation of the CLSI.

Table S11. Activity of various antimicrobials against 52 enterococci isolates cultured from cats with urinary tract infection

	**MIC values (mg/L)**														**S**	**I**	**R**	**n**	**MIC$_{50}$**	**MIC$_{90}$**
	0.00390625	0.0078125	0.015625	0.03125	0.0625	0.125	0.25	0.5	1	2	4	8	16	32	(%)	(%)	(%)		(mg/L)	(mg/L)
Amoxi/Clav						3	5	34	2			1		4	91.8	-	8.2	49	0.5	1
Ampicillin*						1	2	13	27	1		1	4		91.8	-	8.2	49	1	2
Cephalexin										2	1		3	46		IR		52	≥ 32	≥ 32
Cefovecin							2	1	2	1	1	45				IR		52	≥ 8	≥ 8
Chloramphenicol*									1	7	32	5	1	5	90.2	-	9.8	52	4	16
Clindamycin						2		1		4	45					IR		52	≥ 4	≥ 4
Enrofloxacin				1		3	12	26	2		6				84.0	4.0	12.0	50	0.5	4
Erythromycin*						6	1		6	15	10	14			13.5	59.6	27.5	52	2	≥ 8
Gentamicin								1	4	4	5	38				IR		52	≥ 8	≥ 8
Oxacillin										6	46				11.5	-	88.5	52	≥ 4	≥ 4
Penicillin G*					2		1	2	6	28	7	6			100.0	-	-	52	2	≥ 8
Pradofloxacin				1	1	9	27	4	2	6					76	12	12	50	0.25	2
Trim/Sulfa							8			1	43					IR		52	≥ 4	≥ 4
Tetracycline								3	19	2		3	25		46.2	5.8	48.1	52	8	≥ 16

* Indicates that CLSI breakpoints derived from human breakpoints were used. S=susceptible, R=resistant, I= intermediate, IR=intrinsic resistance. The dilution ranges tested are those contained in the white area, and values shown above this range are greater than or equal to the concentration shown. Values at the lower end of these ranges are less than or equal to the lowest concentration tested. Where available, breakpoints are indicated by a vertical line. Grey shaded areas indicate concentration of antimicrobial not tested. Amoxi/Clav, amoxicillin/clavulanic acid (2:1); Trim/Sulfa, trimethoprim-sulfamethoxazole (1:19). MICs were determined using standardized agar dilution methodology based upon the recommendation of the CLSI.

Table S12. Activity of various antimicrobials against 23 coagulase negative staphylococci (CoNS) isolates cultured from cats with urinary tract infection

	**MIC values (mg/L)**														**S**	**I**	**R**	**n**	**MIC_{50}**	**MIC_{90}**
	0.00390625	0.0078125	0.015625	0.03125	0.0625	0.125	0.25	0.5	1	2	4	8	16	32	(%)	(%)	(%)		(mg/L)	(mg/L)
Amoxi/Clav*					18	4	1								100.0	-	-	23	0.0625	0.125
Ampicillin*						18	1	3			1				82.6	13.0	4.3	23	0.125	0.5
Cephalexin								5	18						100.0	-	-	23	1	1
Cefovecin***							22			1					100.0	-	-	23	0.25	0.25
Chloramphenicol**									2	13	7	1			100.0	-	-	23	2	4
Clindamycin				1	3	14	3	2							100.0	-	-	23	0.125	0.25
Enrofloxacin†				4	15	1	2		1						95.7	4.3	-	23	0.0625	0.25
Erythromycin**						1	18	2							100.0	-	-	21	0.25	0.25
Gentamicin**					15	8									100.0	-	-	23	0.06	0.13
Oxacillin**					18	4		1							95.7	-	4.3	23	0.0625	0.125
Penicillin G**					18				1	2		1			81.8	-	18.2	22	0.0625	2
Pradofloxacin†		1	1	5	15			1							95.7	4.3	-	23	0.0625	0.0625
Trim/Sulfa**							22	1			1				95.7	-	4.3	23	0.25	0.25
Tetracycline**					6	12	1	1							100.0			20	0.125	0.125

* Indicates that CLSI breakpoints derived from animal breakpoints were used. ** Indicates that CLSI breakpoints derived from human breakpoints were used. *** Indicates that breakpoints from Fessler et al. (2017) were used [27]. † Indicates that CLSI breakpoints derived from the other animal species (referring to dog and cat) were used. S=susceptible, R=resistant, I= intermediate. The dilution ranges tested are those contained in the white area, and values shown above this range are greater than or equal to the concentration shown. Values at the lower end of these ranges are less than or equal to the lowest concentration tested. Where available, breakpoints are indicated by a vertical line. Grey shaded areas indicate concentration of antimicrobial not tested. Amoxi/Clav, amoxicillin/clavulanic acid (2:1); Trim/Sulfa, trimethoprim-sulfamethoxazole (1:19). MICs were determined using standardized agar dilution methodology based upon the recommendation of the CLSI. Differences between numbers in MIC values and Susceptible/Intermediate/Resistant result from the validation due to oxacillin-resistant isolates.

Table S13. Number of antimicrobial susceptible isolates among cats and dogs suffering from UTI, presented by animal and antibiotic and the most frequently identified species, presenting 96% of all detected bacteria

	**AMC**				**AMP**				**CFX**				**CVF**			
	**dog**		**cat**		**dog**		**cat**		**dog**		**cat**		**dog**		**cat**	
	**n**	**%**	**n**	**%**	**n**	**%**	**n**	**%**	**n**	**%**	**n**	**%**	**n**	**%**	**n**	**%**
CoNS	7	77.8	23	95.8	8	88.9	19	79.2	6	75.0	23	95.8	7	77.8	23	95.8
CoPS	95*	88.8	5*	38.5	50*	49.5*	2	15.4	96*	91.4	7*	53.8	100	96.2	10	83.3
ECC	IR		IR		IR		IR		IR		IR		10	62.5	11	73.3
Enterococcus spp.	60	93.8	45	91.8	61	92.4	45	91.8	IR		IR		IR		IR	
E. coli	315	94.9	148	94.3	257	77.4	120	76.4	314	94.6	152	96.8	321	96.7	150	96.2
Klebsiella spp.	15	8.,3	3	75.0		IR	IR		12	66.7	2	50.0	17	89.5	4	100.0
Proteus spp.	61	96.8	11	100.0	44	72.1	9	81.8	57	93.4	10	90.9	61*	96.8	10*	90.9
Pseudomonas spp.	IR		IR		IR		IR		IR		IR		0	0.0	0	0.0
Sc. canis	45	100.0	6	100.0	45	97.8	7	100.0	44	100.0	6	100.0	46	100.0	7	100.0
**total**	**598**	**94.3**	**241**	**91.3**	**465**	**73.1**	**202**	**77.4**	**529**	**93.6**	**200**	**93.0**	**562**	**93.2**	**215**	**89.2**

	**ENR**				**PRA**				**SXT**				**NIT**			
	**dog**		**cat**		**dog**		**cat**		**dog**		**cat**		**dog**		**cat**	
	**n**	**%**	**n**	**%**	**n**	**%**	**n**	**%**	**n**	**%**	**n**	**%**	**n**	**%**	**n**	**%**
CoNS	7	77.8	22	91.7	7	77.8	22	91.7	9	100.0	22	91.7	1	50.0	15*	100.0
CoPS	95*	92.2	6*	46.2	97*	93.3	6*	46.2	97*	93.3	7*	53.8	79	100.0	7	100.0
ECC	13	81.3	11	73.3	12	75.0	10	66.7	14	87.5	12	80.0	2	28.6	5	41.7
Enterococcus spp.	54	81.8	42	84.0	53	80.3	38	76.0		IR		IR	40	90.9	31	83.8
E. coli	303	91.5	145	92.9	301	90.9	146	93.0	294	88.8	144	91.7	198	92.5	102	91.9
Klebsiella spp.	16	84.2	3	75.0	15	78.9	3	75.0	19	100.0	4	100.0	6	50.0	2	50.0
Proteus spp.	49	77.8	10	90.9	46	73.0	10	90.9	49	77.8	9	81.8	IR		IR	
Pseudomonas spp.	9	64.3	6	50.0	3	21.4	6	50.0	IR		IR		IR		IR	
Sc. canis	41	91.1	7	100.0	41	91.1	7	100.0	45	97.8	7	100.0	32	100.0	5	100.0
**total**	**587**	**86.3**	**252**	**86.3**	**575**	**86.2**	**248**	**84.6**	**527**	**89.6**	**205**	**88.7**	**353**	**91.7**	**167**	**87.4**

* Chi-square test. Indicates significance of the number of susceptible isolates between animal species, (p < 0.05). IR, intrinsic resistance, not validated
